# Granulocyte-Macrophage Colony-Stimulating Factor Inhibition Ameliorates Innate Immune Cell Activation, Inflammation, and Salt-Sensitive Hypertension

**DOI:** 10.3390/cells14151144

**Published:** 2025-07-24

**Authors:** Hannah L. Smith, Bethany L. Goodlett, Gabriella C. Peterson, Emily N. Zamora, Ava R. Gostomski, Brett M. Mitchell

**Affiliations:** Department of Medical Physiology, Texas A&M University College of Medicine, Bryan, TX 77807, USA; hannahsmith@tamu.edu (H.L.S.);

**Keywords:** granulocyte-macrophage colony-stimulating factor, salt-sensitive hypertension, CD38, m1 macrophages, type 2-conventional dendritic cells

## Abstract

Hypertension (HTN) is a major contributor to global morbidity and manifests in several variants, including salt-sensitive hypertension (SSHTN). SSHTN is defined by an increase in blood pressure (BP) in response to high dietary salt, and is associated with heightened cardiovascular risk, renal damage, and immune system activation. However, the role of granulocyte-macrophage colony-stimulating factor (GM-CSF) has not yet been explored in the context of SSHTN. Previously, we reported that GM-CSF is critical in priming bone marrow-derived (BMD)-macrophages (BMD-Macs) and BMD-dendritic cells (BMD-DCs) to become activated (CD38+) in response to salt. Further exploration revealed these cells differentiated into BMD-M1 Macs, CD38+ BMD-M1 Macs, BMD-type-2 conventional DCs (cDC2s), and CD38+ BMD-cDC2s. Additionally, BMD-monocytes (BMDMs) grown with GM-CSF and injected into SSHTN mice traffic to the kidneys and differentiate into Macs, CD38+ Macs, DCs, and CD38+ DCs. In the current study, we treated SSHTN mice with an anti-GM-CSF antibody (aGM) and found that preventive aGM treatment mitigated BP, prevented renal inflammation, and altered renal immune cells. In mice with established SSHTN, aGM treatment attenuated BP, reduced renal inflammation, and differentially affected renal immune cells. Adoptive transfer of aGM-treated BMDMs into SSHTN mice resulted in decreased renal trafficking. Additionally, aGM treatment of BMD-Macs, CD38+ BMD-M1 Macs, BMD-DCs, and CD38+ BMD-cDC2s led to decreased pro-inflammatory gene expression. These findings suggest that GM-CSF plays a role in SSHTN and may serve as a potential therapeutic target.

## 1. Introduction

Hypertension (HTN) is characterized by chronically elevated blood pressure (BP) and is often associated with immune system activation and inflammation [1]. Salt-sensitive HTN (SSHTN) occurs when individuals experience an increase in BP in response to increased dietary salt intake [2,3]. Among the 122.4 million adults in the United States with HTN, approximately 51% are estimated to have SSHTN, while 26% of normotensive individuals also exhibit salt sensitivity [4,5,6,7]. SSHTN is associated with an increased risk of severe cardiovascular events, such as heart failure and chronic kidney disease, due to sustained high BP, inflammation, and sodium retention [8,9,10]. Despite available treatment options, only 22.5% of hypertensive adults have their BP under control [11]. As many existing treatments do not consider the role of the immune system, a more thorough understanding of the specific immune mechanisms and immune cell involvement in SSHTN may aid in the development of more targeted and personalized therapies.

Traditionally, HTN has been attributed to factors such as high-salt intake, elevated angiotensin II or aldosterone levels, and increased sympathetic tone. These mechanisms do not act independently, but rather cohesively work together, along with contributions from immune cells and other factors [12]. This broader understanding has led to the exploration of additional contributors, including growth factors like granulocyte-macrophage colony-stimulating factor (GM-CSF). GM-CSF is primarily recognized as a hematopoietic growth factor due to its role in the proliferation and differentiation of bone marrow progenitor cells [13]. However, its functions extend beyond hematopoiesis, as it plays a significant role in inflammatory autoimmune conditions, including rheumatoid arthritis and giant cell arteritis [13,14,15]. Blocking GM-CSF or its receptor in these conditions have been reported to reduce inflammation, alleviate pain, and decrease immune cell infiltration [13,14,15]. Notably, a patient with pulmonary alveolar proteinosis has been successfully treated with inhaled recombinant GM-CSF to restore GM-CSF-dependent macrophage (Mac) functions, including surfactant clearance; however, elevated GM-CSF levels were linked to transient pulmonary HTN [16]. Additionally, GM-CSF levels are elevated in the serum of hypertensive patients, with even higher levels observed in those with concurrent inflammatory conditions [17,18]. Despite its established role in chronic inflammation and its ability to activate and recruit myeloid immune cells to sites of inflammation, GM-CSF has not been studied in the context of SSHTN to our knowledge [19]. Given its pro-inflammatory and immune-regulatory effects, understanding GM-CSF’s role in SSHTN may provide new insights into the mechanisms underlying HTN and aid in the development of new therapeutic options for patients.

The accumulation of activated immune cells in the kidneys has been documented in various mouse models of SSHTN, and we have found specifically that cluster of differentiation 38 (CD38)+ Macs and dendritic cells (DCs) are increased [20,21,22,23,24,25,26,27,28,29]. CD38 is a marker of activation, and cell surface expression promotes pro-inflammatory signaling in Macs and DCs either via its ectoenzymatic properties or interactions with other receptors [30,31,32]. CD38 functions as both an activation marker and a regulator of intracellular signaling, influencing calcium flux, nicotinamide adenine dinucleotide (NAD+) metabolism, and immune cell function [33]. Notably, salt exposure has been shown to drive CD38 expression on Macs and DCs, further enhancing their pro-inflammatory potential and ability to secrete GM-CSF [34].

Both Macs and DCs, when activated under hypertensive conditions, are prominent producers of tumor necrosis factor alpha (TNFα), interleukin (IL)-6, and IL-1β, which contribute to vascular damage and inflammation [12]. Pro-inflammatory Macs, traditionally referred to as M1 Macs, play a prominent role in SSHTN by promoting inflammation, contributing to vascular damage, and enhancing renal sodium retention [35]. High salt has been found to potentiate lipopolysaccharide-induced activation of M1 Macs through the p38/cFos and/or Erk1/2/cFos pathways, while simultaneously suppressing anti-inflammatory pathways that would promote IL-4-induced Mac polarization [36]. Salt activates DCs via accumulation of isoketals or formation of isolevuglandin adducts, the production of superoxide, and epithelial sodium channels [37,38,39]. Type-2 conventional DCs (cDC2s), an understudied DC subtype in the context of SSHTN, present antigens via MHCII to activate CD4+ helper T cells and produce cytokines and chemokines to recruit and activate innate immune cells [40]. Interestingly, we have observed an increase in renal CD38+ Macs, CD38+ M1 Macs, CD38+ DCs, and CD38+ cCD2s in mice with SSHTN as well as in vitro after treating GM-CSF grown bone marrow derived monocytes (BMDMs) with salt [29]. Both CD38+ M1 Macs and CD38+ cDC2s represent key immune cell populations involved in the pathogenesis of SSHTN, highlighting them as potential targets for therapeutic intervention.

We recently reported that GM-CSF is a necessary component in priming cells to respond to salt and enter an activated CD38+ state [29]. We also found that adoptively transferred GM-CSF grown BMDMs trafficked more effectively to the kidneys in mice with SSHTN, and more of these cells became CD38+ Macs and DCs [29]. The infiltration of these cells into the kidneys of SSHTN mice suggests that they play a direct role in renal dysfunction, raising the possibility that targeting GM-CSF could mitigate SSHTN by limiting renal accumulation of CD38+ M1 Macs and CD38+ cDC2s. In this study, we hypothesized that inhibiting GM-CSF in mice with SSHTN would prevent BP elevation, renal inflammation, and renal dysfunction, while limiting increases in CD38+ M1 Macs and CD38+ cDC2s. Additionally, we hypothesized that BMDMs treated with an anti-GM-CSF antibody (aGM) would be less likely to differentiate into CD38+ immune cells or traffic to SSHTN kidneys following adoptive transfer. Finally, we hypothesized that salt treatment would increase pro-inflammatory gene expression in CD38+ M1 Macs and CD38+ cDC2s, which could be inhibited by aGM treatment.

## 2. Materials and Methods

### 2.1. Animal Models

All experimental procedures involving animals were conducted with prior authorization obtained on 29 April 2022, from the Texas A&M University Institutional Animal Care and Use Committee (IACUC: 2022-0083) and were performed in accordance with the guidelines outlined in the NIH Guide for the Care and Use of Laboratory Animals. Male and female wild-type C57BL/6J mice aged 8–10 weeks were acquired from Jackson Laboratories (Bar Harbor, ME, USA) and given 2 weeks to acclimate to our facility.

SSHTN was first induced via administration of nitro-l-arginine methyl ester hydrochloride (L-NAME; 0.5 mg/mL; Sigma, St. Louis, MO, USA) in drinking water for 2 weeks. This was followed by a 2-week washout period with normal drinking water to bring BP back to normal [41,42]. At the end of the washout period, the mice were given a 4% high-salt diet (Teklad Envigo, Huntingdon, UK) until the conclusion of their assigned model. For all mouse groups, drinking water and diets were available at all times. The total amount of drinking water and diet consumed by each mouse was not tracked. When each model concluded, mice were euthanized by perfusion under 5% inhaled isoflurane and death was confirmed via cervical dislocation.

#### 2.1.1. Preventive Anti-Granulocyte-Macrophage Colony-Stimulating Factor Treatment

To determine if aGM treatment could prevent the increase in systolic blood pressure (SBP) associated with SSHTN, female mice received a 4% high-salt diet for 1 week. Beginning 3 days prior to diet administration, mice received daily intraperitoneal injections of either anti-mouse GM-CSF (aGM; 300 µg/mL; Bio X Cell, Lebanon, NH, USA) (SSHTN + aGM; *n* = 4–5) or rat IgG2a isotype control antibody, anti-trinitrophenol (CAB; 300 µg/mL; Bio X Cell) (SSHTN + CAB; *n* = 3–5) [43]. Beginning on Day 4 of the high-salt diet phase, mice received these injections twice daily at 12 h intervals until they were euthanized.

#### 2.1.2. Therapeutic Anti-Granulocyte-Macrophage Colony-Stimulating Factor Treatment

To determine if aGM treatment could attenuate SBP in established SSHTN, male mice received a 4% high-salt diet for 3 weeks. After 3 days of a high-salt diet, mice received injections of either aGM (SSHTN + aGM; *n* = 4–5) or CAB (SSHTN + CAB; *n* = 4–5) as described above. These injections were administered every 3 days throughout the 3-week model.

### 2.2. Systolic Blood Pressure Measurements

During the treatment period of the preventive group, SBP was recorded every other day. SBP was recorded weekly for the duration of the therapeutic treatment group. SBP was recorded via tail cuff using the IITC Life Science non-invasive BP acquisition system (IITC Inc., Woodland Hills, CA, USA). Mice were acclimated to the tail-cuff procedure over a minimum of 3 consecutive days prior to initial measurements. Before each session, mice were placed in a quiet room for 30 min to reduce stress, followed by placement in pre-warmed restrainers inside the 34 °C warming chamber. An additional 10 min acclimation period in the restrainers was provided prior to recording the first SBP trace. All measurements were conducted by two independent observers blinded to experimental conditions.

### 2.3. Serum and Urine Collection and Measures

Mice were first acclimated to single-capacity metabolic cages (Hatteras, Cary, NC, USA) for 24 h. After this period, fresh urine collection tubes were placed in the cages, and urine was continuously collected over the subsequent 24 h, preceding euthanasia. Blood was obtained from the right ventricle, and serum was isolated and stored alongside urine samples at −80 °C until analysis. Serum and urine sodium concentrations were quantified using capillary electrophoresis on a DxC 700 AU Chemistry Analyzer (Beckman Coulter, Brea, CA, USA). Creatinine concentrations from both serum and urine were measured via direct potentiometry using a P/ACE MDQ Plus Capillary Electrophoresis System (Sciex, Redwood City, CA, USA). These measurements were used to calculate fractional excretion of sodium (FENa) and creatinine clearance. FENA was calculated with this formula:

$$\mathrm{FENA}\left(\%\right)=\frac{\mathrm{Urine}\;\mathrm{Sodium}\;\mathrm{Concentration}\;\left(\frac{\mathrm{mEq}}{L}\right)\times \mathrm{Plasma}\;\mathrm{Creatinine}\;\mathrm{Concentration}\;\left(\frac{\mathrm{mg}}{\mathrm{dL}}\right)}{\mathrm{Plasma}\;\mathrm{Sodium}\;\mathrm{Concentration}\;\left(\frac{\mathrm{mEg}}{L}\right)\times \mathrm{Urine}\;\mathrm{Creatine}\;\mathrm{Clearance}\;(\frac{\mathrm{mg}}{\mathrm{dL}})}\times 100$$

Creatinine clearance was calculated with this formula:

$$\mathrm{Creatinine}\;\mathrm{Clearance}\;\left(\frac{\mathrm{mL}}{\min}\right)=\frac{\mathrm{Urine}\;\mathrm{Creatinine}\;\left(\frac{\mathrm{mg}}{\mathrm{dL}}\right)\times \mathrm{Urine}\;\mathrm{Volume}\;\left(\mathrm{mL}\right)}{\mathrm{Plasma}\;\mathrm{Creatinine}\;\left(\frac{\mathrm{mg}}{\mathrm{dL}}\right)\times \mathrm{Time}\;(\min)}\;$$

### 2.4. In Vitro Cell Culture

Mice were euthanized via extended exposure to 5% inhaled isoflurane, with death confirmed through cervical dislocation. Following euthanasia, the entire mouse was doused in 70% ethanol and transferred to a laminar flow hood for aseptic dissection and harvest of the femur and tibia bones. The protocol described by Smith et al. was used to isolate BMDMs [29]. BMDMs were seeded in 6-well plates at 2.5 × 10^6^–3 × 10^6^ and differentiated into BMD-Macs or BMD-DCs based upon different media conditions. To generate BMD-Macs, cells were cultured in DMEM high-glucose media (Thermo Fisher Scientific, Waltham, MA, USA) with 10% heat-inactivated FBS (Sigma) and 1% penicillin/streptomycin (Thermo Fisher Scientific) [44]. To generate BMD-DCs, cells were cultured in RPMI 1640 (Thermo Fisher Scientific) with 10% FBS, 1 mM HEPES (Cytiva Life Sciences, Marlborough, MA, USA), 1% penicillin/streptomycin, and 50 µM 2-mercaptoethanol (Sigma) [45].

BMD-Macs and BMD-DCs were cultured for a total of 7 days. On Day 0, cells were plated in 3 mL of either Mac or DC-specific media with 25 ng/mL of GM-CSF (R&D Systems, Minneapolis, MN, USA) and incubated at 37 °C with 5% CO_2_. For BMD-Macs, complete media replacements took place on Days 3 and 6. The 24 h treatment of aGM (1.2 µg/mL) and salt (180 mM; Sigma) took place on Day 6 after the media change was completed. For BMD-DCs, complete media replacements was performed on Days 2, 4, and 6. The 24 h treatment of aGM (1.2 µg/mL) and salt (180 mM) took place on Day 6 after the media change was completed. Salt or mannitol was added to the base media to achieve a final concentration of 180 mM. Normal salt (control), mannitol, and high salt (180 mM) conditions were used to assess the effects of osmolarity and ionic activation on BMD-Macs and BMD-DCs grown in GM-CSF (Supplementary Figure S1A–I).

#### Anti-Granulocyte-Macrophage Colony-Stimulating Factor Treatment

On Day 6, cells were treated with aGM for 30 min prior to the addition of GM-CSF and salt to bring the media concentration to 180 mM for a 24 h treatment period (aGM + SALT). Control cells received aGM for 30 min prior to the addition of GM-CSF and were not treated with salt (aGM + CONTROL). After 24 h of treatment, cells were harvested on Day 7 using TrypLE (Thermo Fisher Scientific). Harvested cells were used for adoptive transfer, flow cytometry, cell sorting, or real-time quantitative polymerase chain reaction (qRT-PCR).

### 2.5. Adoptive Transfer

On Day 7 of cell culture, control BMDMs were grown in GM-CSF (SSHTN + GM; *n* = 4) or BMDMs treated with aGM (SSHTN + aGM; *n* = 4) were harvested and incubated with CellTracker™ Deep Red dye (Thermo Fisher Scientific) for 45 min at 37 °C. Next, 1 × 10^6^ live CellTracker+ cells were injected intraperitoneally into SSHTN mice. Mice were euthanized 12 h after the adoptive transfer and kidneys were isolated for flow cytometry.

### 2.6. Flow Cytometry

Immediately following euthanasia, kidneys were harvested and processed for flow cytometry as described by Smith et al. [29]. Isolated kidney cells were stained with either viability dye Ghost Dye Red 710 (Tonbo Biosciences, San Diego, CA, USA) or Ghost Dye Violet 510 (Tonbo Biosciences). To prevent non-specific binding, cells were incubated with anti-mouse CD16/CD32 Fc block (BD Biosciences, San Jose, CA, USA). Surface staining was performed using fluorophore-conjugated antibody panels, with kidney samples stained at a 1:100 antibody dilution (Supplementary Table S1A–C). Immune cells isolated from in vitro cultures were harvested as described previously and stained using the same antibody panels at a 1:200 dilution. Data acquisition was carried out on Cytek Aurora 5L (Cytek Biosciences, Fremont, CA, USA) and analysis was conducted with FlowJo v10.8 (FlowJo, LLC, Ashland, OR, USA). Results are expressed as a percent of CD45+ cells or number of cells. Gating strategies were established by referencing unstained specimens and compensation controls (Supplementary Figure S2A–C).

### 2.7. Cell Sorting

On Day 7 of culture, BMD-Macs and BMD-DCs were harvested and stained as described previously with antibody panels optimized for cell sorting (Supplementary Table S1D,E). CD38+ BMD-M1 Macs and CD38+ BMD-cDC2s were sorted from both conditions using a BD FACSAria™ III Cell Sorter (BD Biosciences; Supplementary Figure S2D–E). Cells were sorted into tubes that had been pre-coated with PBS and 20% FBS, then emptied before sorting into TRI Reagent (Zymo Research, Irvine, CA, USA) for RNA isolation.

### 2.8. Quantitative Real-Time Reverse Transcription Polymerase Chain Reaction

Decapsulated kidneys were rapidly frozen in liquid nitrogen and stored at −80 °C. Total RNA was extracted from kidney tissue using the Quick-RNA MiniPrep Kit (Zymo Research, according to the manufacturer’s guidelines. Following BMDM harvest, RNA from BMD-Macs and BMD-DCs was similarly isolated using the Direct-zol RNA MiniPrep Kit (Zymo Research) according to the manufacturer’s protocol. In accordance with the manufacturers protocol, approximately 1 µg of RNA was reverse transcribed into cDNA using the RT^2^ First Strand Kit (Qiagen, Germantown, MD, USA). qRT-PCR reactions (10 µL total volume) were prepared by combining PowerUp SYBR Green Master Mix (Applied Biosystems, Waltham, MA, USA), nuclease-free water (Invitrogen, Waltham, MA, USA), gene-specific primers (10 μM; Sigma), and cDNA from the relevant sample. RPS18 served as the endogenous control. Quantification of mRNA expression was performed on a QuantStudio6 Flex Real-Time PCR System (Applied Biosystems), and gene expression was determined by calculating fold changes using the 2−ΔΔCT method. Primer sequences were designed using the NCBI Gene Database and are listed in Supplementary Table S2.

### 2.9. Immunofluorescence Staining

Kidneys were fixed in 4% paraformaldehyde (PFA; Sigma) at 4 °C for 24 h, then rinsed with DPBS, embedded in paraffin, and sagittally sectioned at a thickness of 5 μm. Sections were subsequently deparaffinized, rehydrated, and permeabilized with 0.1% Triton X-100 (Bio-Rad, Hercules, CA, USA). To minimize non-specific antibody binding, tissue sections were incubated with 10% AquaBlock (EastCoastBio, North Berwick, ME, USA) for 1 h at room temperature. Overnight immunostaining was performed at 4 °C with alpha-smooth muscle actin (rabbit polyclonal; 1:100; Invitrogen) to visualize vascular smooth muscle cells. The next day, sections were washed and incubated with Alexa Fluor 488–conjugated secondary antibodies (goat anti-rabbit; 1:250; Invitrogen) for 1 h at room temperature. Slides were mounted with ProLong Gold Antifade Mountant containing DAPI (Invitrogen). The number of lumen-forming blood vessels was quantified by two independent, blinded investigators, using an Olympus BX51 fluorescence microscope equipped with a DP72 camera (Olympus, Tokyo, Japan), and results were averaged.

### 2.10. Statistical Analysis

All statistical analyses were performed using GraphPad Prism v8.4.3 (GraphPad Software, Inc., Boston, MA, USA). Comparisons between groups were made using two-tailed unpaired Student’s *t*-tests. Data are presented in bar graphs displaying mean ± SEM or mean ± SD, with individual data points shown as dots. Statistical significance was defined at *p* < 0.05 with an asterisk (*).

## 3. Results

### 3.1. Preventive Granulocyte-Macrophage Colony-Stimulating Factor Inhibition Attenuated Systolic Blood Pressure, Inflammation, and Immune Cell Activation in Mice with Salt-Sensitive Hypertension

**Figure 1:**
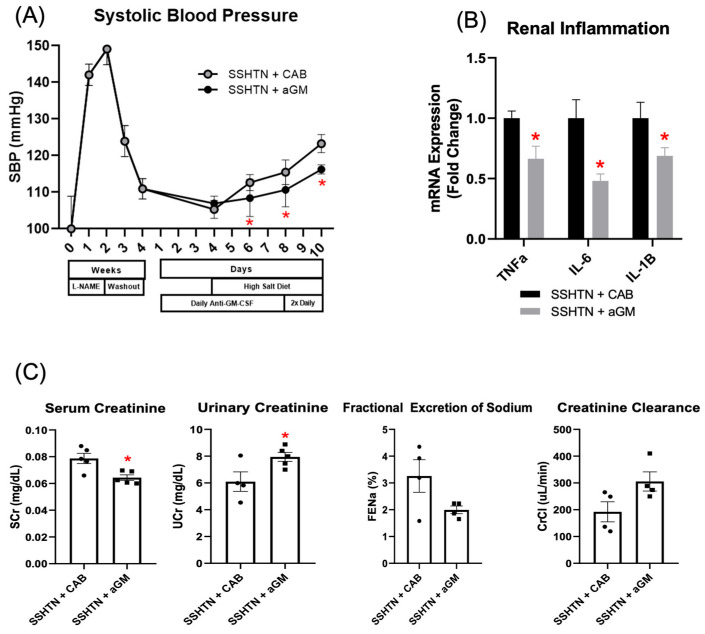
Inhibition of granulocyte-macrophage colony-stimulating factor prior to the induction of salt-sensitive hypertension attenuated systolic blood pressure, lowered renal inflammatory gene expression, and mitigated renal function. (**A**) SBP measurements in CAB-treated and aGM-treated SSHTN mice (*n* = 4–5). BP was recorded weekly for four weeks and every other day for the final 7 days using the tail-cuff method. (**B**) Renal expression of inflammation-related genes in CAB-treated and aGM-treated SSHTN mice (*n* = 4–5). (**C**) Serum creatinine and urinary creatinine concentrations, FENa, and creatinine clearance in CAB-treated and aGM-treated SSHTN mice (*n* = 4–5). BP data is presented as the mean ± SD and statistical analyses were performed with an unpaired Student’s *t*-test, * *p* < 0.05 vs. SSHTN + CAB. All other data are presented as the mean ± SEM and statistical analyses were performed with an unpaired Student’s *t*-test, * *p* < 0.05 vs. SSHTN + CAB.

GM-CSF has been found to play a prominent role in inflammatory conditions; however, its role in SSHTN remains unexplored. In this study, we tested the effectiveness of aGM as a preventive treatment before the induction of SSHTN. Mice began receiving aGM treatment 3 days before the administration of a high-salt diet. They received one injection daily, which was increased to two injections per day during the final three days of the model. SBP was measured weekly before diet initiation and then every other day after treatment began. SSHTN mice treated with aGM exhibited a blunted increase in SBP compared to those treated with the CAB (Figure 1A). After 10 days of injections, when SBP began to rise, mice were euthanized to assess inflammatory responses, immune phenotypes, and renal function.

To assess inflammation, we measured renal mRNA expression of pro-inflammatory genes commonly associated with SSHTN, including *Tnfα, Il-6*, and *Il-1β* [12,46]. Preventive aGM treatment significantly decreased renal expression of these genes in SSHTN mice (Figure 1B). aGM-treated mice exhibited decreased serum creatinine and increased urinary creatinine, indicating protected kidney function (Figure 1C). This was further supported by a trend toward increased creatinine clearance; however, FENa was not changed significantly (Figure 1C).

**Figure 2:**
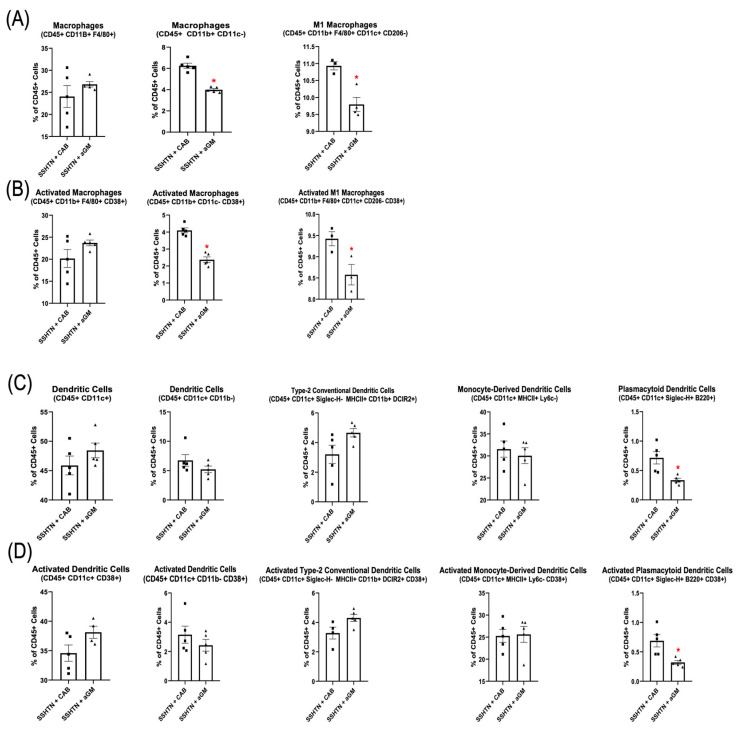
Preventive anti-granulocyte-macrophage colony-stimulating factor treatment mitigated renal macrophage and dendritic cell differentiation in salt-sensitive hypertension mice. (**A**) Flow cytometry data assessing Mac populations, as well as (**B**) their CD38+ counterparts, along with (**C**) DC populations and (**D**) their CD38+ counterparts in CAB-treated and aGM-treated SSHTN mice (*n* = 3–5). Data are presented as mean ± SEM and statistical analyses were performed with an unpaired Student’s *t*-test, * *p* < 0.05 vs. SSHTN + CAB.

To evaluate the impact of aGM on renal immune cells, we performed flow cytometry to characterize renal Mac and DC subsets. Renal Macs (CD45+ CD11b+ CD11c−) and CD38+ Macs (CD45+ CD11b+ CD11c− CD38+) were decreased significantly in SSHTN mice treated with aGM (Figure 2A,B). Renal M1 Macs (CD45+ CD11b+ F4/80+ CD11c+ CD206−) and CD38+ M1 Macs were also decreased significantly (Figure 2A,B). Plasmacytoid DCs (pDCs; CD45+ CD11c+ Siglec–H+ B220+), and CD38+ pDCs were decreased significantly in SSHTN + aGM mice (Figure 2C,D). Preventive treatment with aGM induced distinct effects on renal Mac and DC subsets, with some subsets showing decreased populations and others unaffected.

### 3.2. Anti-Granulocyte-Macrophage Colony-Stimulating Factor Treatment Decreased Systolic Blood Pressure, Decreased Inflammation, and Differentially Affected Renal Immune Cell Populations in Mice with SSHTN

**Figure 3:**
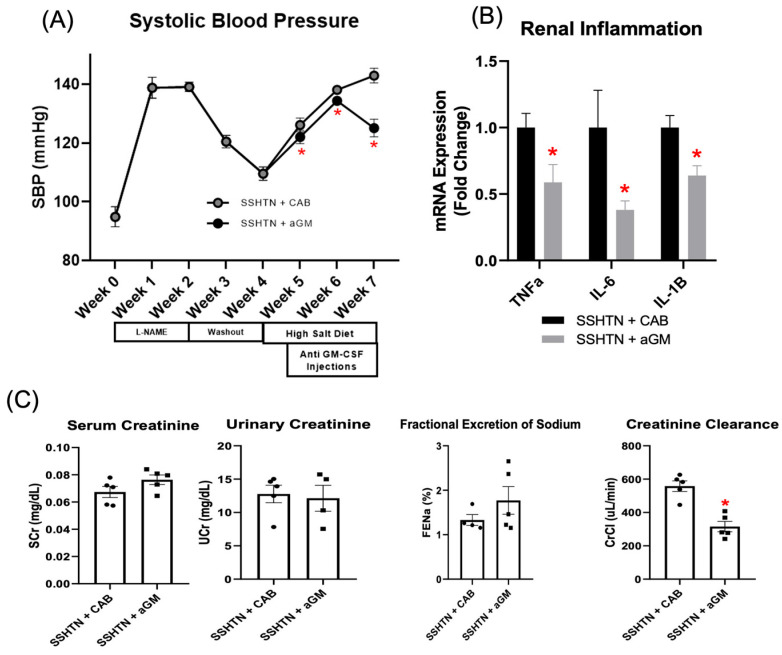
Treatment with anti-granulocyte-macrophage colony-stimulating factor attenuated systolic blood pressure, reduced renal inflammation, and decreased creatinine clearance in mice with established salt-sensitive hypertension. (**A**) SBP measurements in CAB-treated and aGM-treated SSHTN mice (*n* = 4–5). BP was recorded weekly via tail-cuff method. (**B**) Renal expression of inflammation-related genes in CAB-treated and aGM-treated SSHTN mice (*n* = 4–5). (**C**) Serum creatinine and urinary creatinine concentrations, FENa, and creatinine clearance in CAB-treated and aGM-treated SSHTN mice (*n* = 4–5). BP data is presented as the mean ± SD and statistical analyses were performed with an unpaired Student’s *t*-test, * *p* < 0.05 vs. SSHTN + CAB. All other data are presented as the mean ± SEM and statistical analyses were performed with an unpaired Student’s *t*-test, * *p* < 0.05 vs. SSHTN + CAB.

We next investigated the effects of inhibiting GM-CSF in established SSHTN. SSHTN mice received CAB or aGM injections 3 days after the start of a high-salt diet, and every 3 days for 3 weeks. SBP remained significantly decreased throughout the treatment period, with a notable decrease at Week 7 (Figure 3A). qRT-PCR revealed significant reductions in renal gene expression of *Tnfα*, *Il-6*, and *Il-1β* in SSHTN mice treated with aGM compared to those receiving the CAB (Figure 3B). Though serum creatinine, urine creatinine, and FENa were unchanged by aGM treatment, creatinine clearance was decreased significantly in SSHTN + aGM mice (Figure 3C).

**Figure 4:**
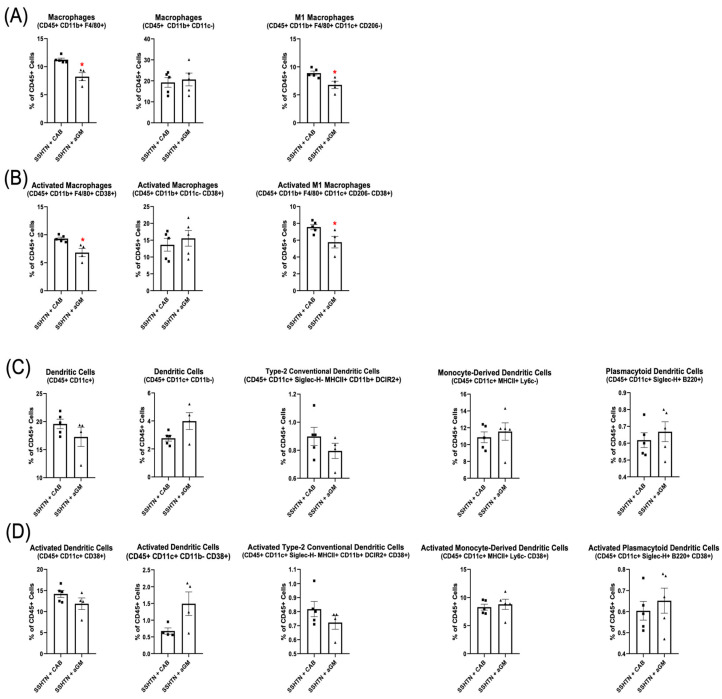
Anti-granulocyte-macrophage colony-stimulating factor treatment differentially affected renal Mac and DC subtypes. Renal flow cytometry data assessing (**A**) Mac populations, as well as their (**B**) CD38+ counterparts, along with (**C**) DC populations and (**D**) their CD38+ counterparts in CAB-treated and aGM-treated SSHTN mice (*n* = 4–5). Data are presented as the mean ± SEM and statistical analyses were performed with an unpaired Student’s *t*-test, * *p* < 0.05 vs. SSHTN + CAB.

Flow cytometry analysis of renal immune cell populations demonstrated a significant decrease in Macs (CD45+ CD11b+ F4/80+) and M1 Macs in SSHTN + aGM mice (Figure 4A). Additionally, renal CD38+ Macs (CD45+ CD11b+ F4/80– CD38+) and CD38+ M1 Macs were reduced significantly in aGM-treated SSHTN mice (Figure 4B). However, aGM treatment after SSHTN onset was less effective at decreasing DC and activated DC populations (Figure 4C,D). Overall, starting aGM treatment after the induction of SSHTN led to reductions in SBP and renal inflammation, while differentially affecting renal function and immune cell populations, with Macs being the most notably changed.

### 3.3. Inhibiting Granulocyte-Macrophage Colony-Stimulating Factor Reduced Recruitment and Differentiation of Immune Cells in the Kidneys of Mice with Salt-Sensitive Hypertension

A hallmark of SSHTN is immune cell trafficking to the kidneys, which promotes inflammation and tissue damage and further contributes to the progression and associated complications of HTN. Previously, we determined that BMDMs cultured with GM-CSF preferentially traffic to the kidneys in SSHTN mice [29].

**Figure 5:**
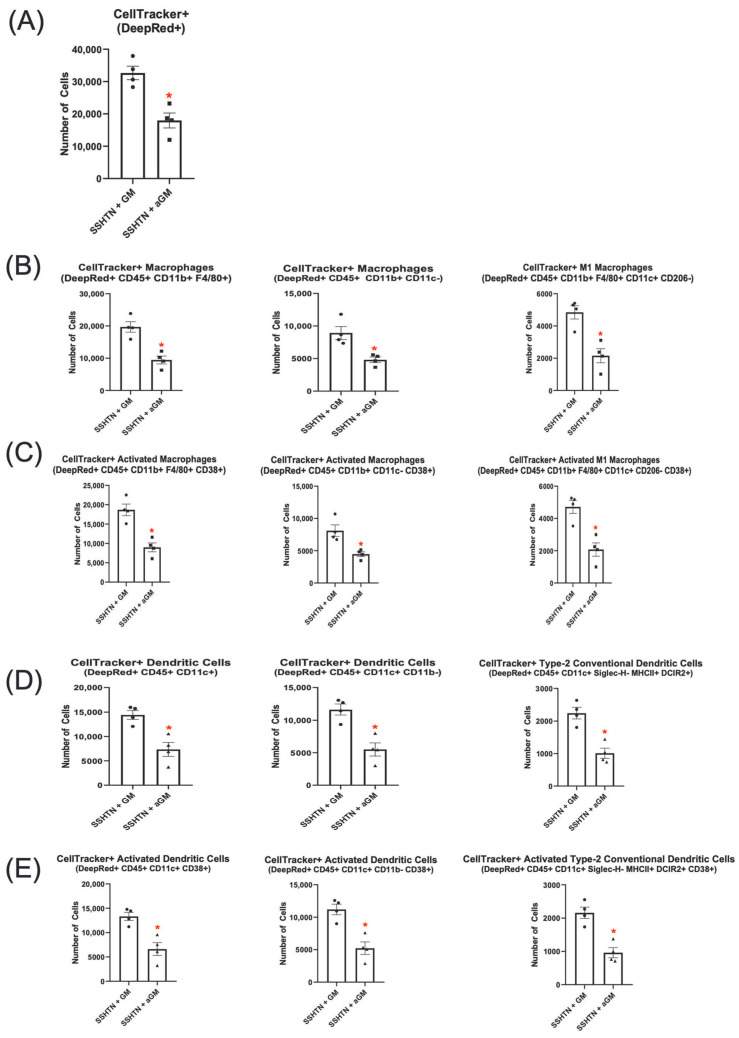
Adoptive transfer of anti-granulocyte-macrophage colony-stimulating factor-treated bone marrow-derived monocytes into salt-sensitive hypertension mice reduced macrophage and dendritic cell differentiation. Flow cytometry data assessing the number of (**A**) CellTracker+ cells grown with GM-CSF or treated with aGM that trafficked to SSHTN kidneys (*n* = 4). The CellTracker+ cells differentiated into (**B**) Mac populations, (**C**) CD38+ Macs, (**D**) DC populations and (**E**) CD38+ DCs. Data are presented as the mean ± SEM and statistical analyses were performed with an unpaired Student’s *t*-test, * *p* < 0.05 vs. SSHTN + GM.

To investigate whether immune cell trafficking to the kidneys could be reduced, we adoptively transferred BMDMs treated with aGM into SSHTN mice. We found that significantly fewer CellTracker+ aGM-treated cells migrated to the kidneys in mice with SSHTN (Figure 5A). Additionally, fewer CellTracker+ aGM-treated cells differentiated into Macs (CD45+ CD11b+ F4/80+ and CD45+ CD11b+ CD11c−) and CD38+ Macs within the kidneys (Figure 5B,C). Similarly, fewer of these cells differentiated into M1 Macs and CD38+ M1 Macs. 

CellTracker+ DC subsets were also analyzed, revealing significantly fewer aGM-treated cells that differentiated into CellTracker+ DCs (CD45+ CD11c+ and CD45+ CD11c+ CD11b–) or their CD38+ subsets within SSHTN kidneys compared to GM-CSF-treated cells (Figure 5D,E). Further analysis showed a significant decrease in CellTracker+ cDC2s and CD38+ cDC2s from the aGM-treated cells (Figure 5D,E).

Together, these findings suggest that blocking GM-CSF reduced recruitment and differentiation of Macs, CD38+ Macs, M1 Macs, CD38+ M1 Macs, DCs, CD38+ DCs, cDC2s, and CD38+ cDC2s in the kidneys of SSHTN mice. These results are indicative of the critical role GM-CSF plays in SSHTN by promoting renal immune cell trafficking, differentiation, and activation.

### 3.4. Inhibition of Granulocyte-Macrophage Colony-Stimulating Factor Decreased Macrophage and Dendritic Cell Polarization and Pro-Inflammatory Gene Expression

The immune system plays a key role in the cardiovascular-renal complications associated with HTN, and we have observed CD38+ M1 Macs and CD38+ cDC2s increased in the kidneys of mice with SSHTN [29]. However, the profile and impact of CD38+ M1 Macs and CD38+ cDC2s in SSHTN has yet to be determined. Previously, we established that GM-CSF is necessary in vitro when treating BMDMs with salt to replicate renal immune cell phenotypes observed in SSHTN [29]. To further investigate, we examined whether inhibiting GM-CSF in vitro prior to salt treatment would reduce the abundance of these immune cell populations and decrease their phenotypic characteristics.

**Figure 6:**
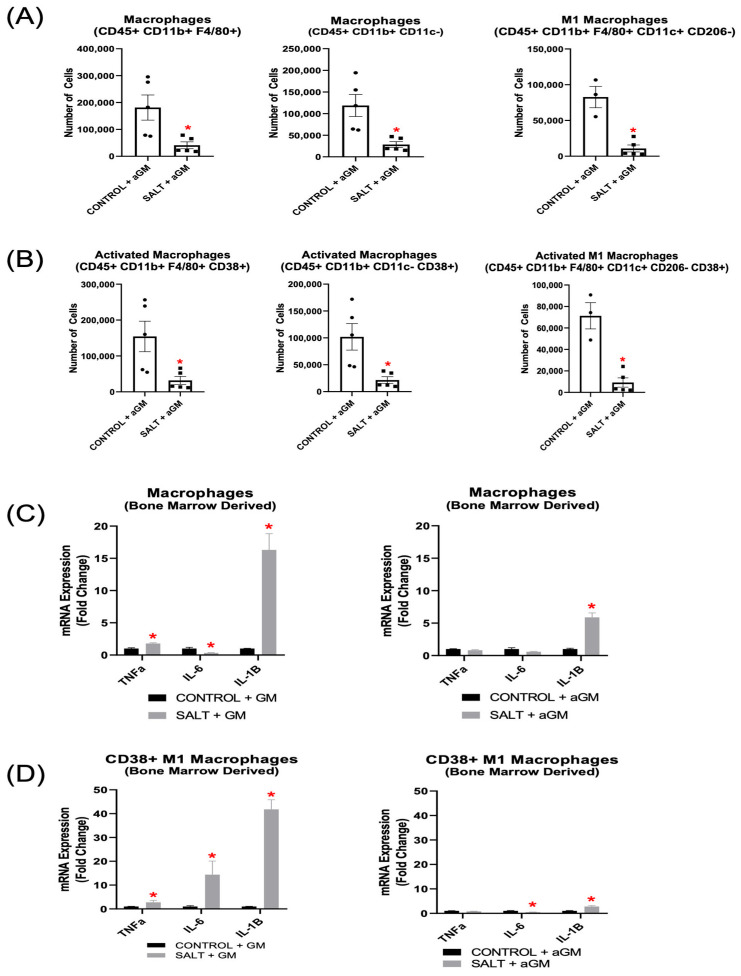
Anti-granulocyte-macrophage colony-stimulating factor inhibition decreased bone marrow-derived Macrophage differentiation and pro-inflammatory gene expression. Flow cytometry analysis of (**A**) BMD-Macs and (**B**) CD38+ BMD-Macs treated with aGM prior to salt (*n* = 3–5). Cellular expression of inflammation-related genes in BMD-Macs (**C**) grown with GM-CSF (GM) and treated with salt or BMD-Macs treated with aGM prior to salt. (**D**) CD38+ M1 Macs were sorted from either BMD-Macs grown with GM-CSF and salt or BMD-Macs treated with aGM and salt to analyze cellular expression of pro-inflammatory genes. Data are presented as the mean ± SEM and statistical analyses were performed with an unpaired Student’s *t*-test, * *p* < 0.05 vs. Control + GM or Control + aGM.

Our results demonstrate that inhibiting GM-CSF prior to salt treatment decreased BMD-Macs, along with their respective CD38+ subsets (Figure 6A,B). Notably, BMD-M1 Macs, including CD38+ BMD-M1 Macs, were also reduced significantly (Figure 6A,B). Further analysis of unsorted BMD-Macs revealed that when cultured with GM-CSF and exposed to salt, *Tnfα* and *IL-1β* gene expression were increased significantly, and *Il-6* expression was decreased significantly. However, when BMD-Macs were treated with aGM before salt exposure, *Tnfα* expression returned to baseline levels, *Il-6* remained decreased, and *Il-1β*, although still elevated, exhibited a lower fold change compared to GM-CSF-treated cells (Figure 6C). Additionally, when BMDMs were differentiated into BMD-Macs with GM-CSF, then exposed to salt and sorted for CD38+ BMD-M1 Macs (CD11b+ CD11c+ CD206− CD38+), they exhibited significantly increased expression of *Tnfα*, *Il-6*, and *Il-1β* (Figure 6D). However, aGM treatment before salt exposure restored *Tnfα* expression to baseline, significantly reduced *Il-6* gene expression, and blunted—but did not fully normalize *Il-1β* expression (Figure 6D).

**Figure 7:**
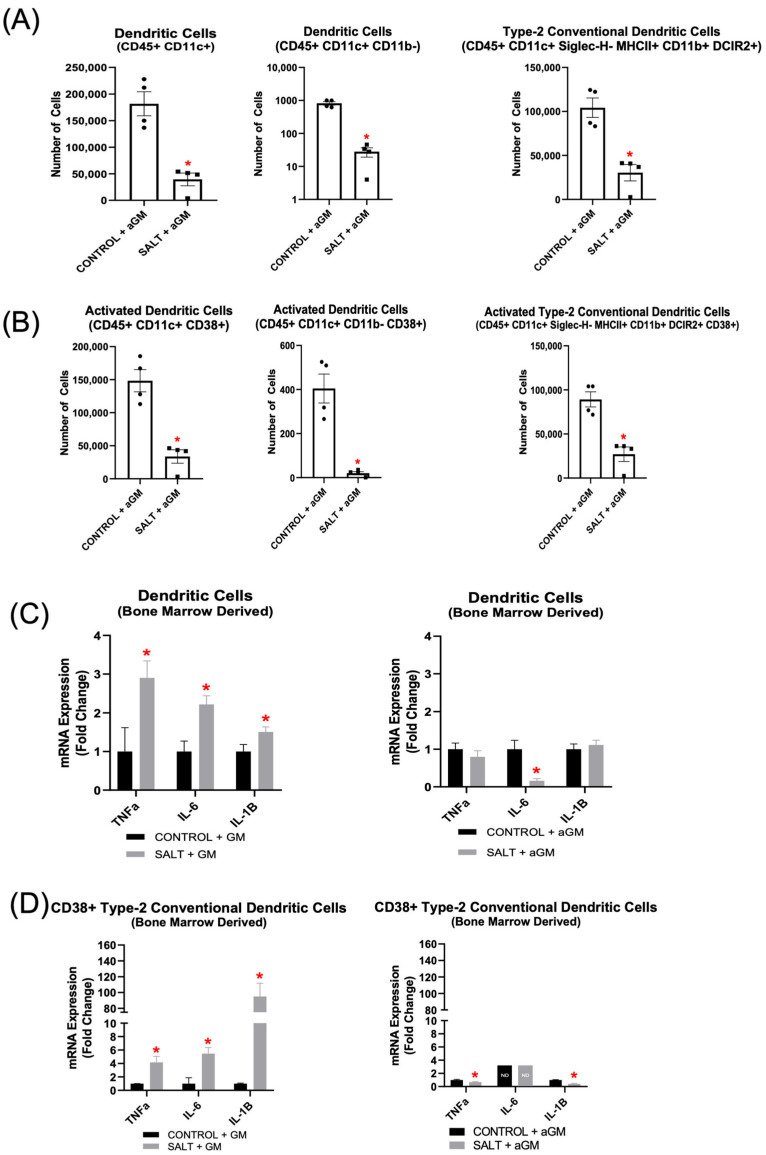
Anti-granulocyte-macrophage colony-stimulating factor inhibition decreased bone marrow-derived dendritic cell differentiation and inflammatory gene expression. Flow cytometric analysis of (**A**) BMD-DCs and (**B**) CD38+ BMD-DCs treated with aGM prior to salt stimulation (*n* = 4–5). Cellular expression of inflammation-related genes in BMD-DCs (**C**) grown with GM-CSF (GM) and treated with salt or BMD-DCs treated with aGM prior to salt. (**D**) CD38+ cDC2s were sorted from either BMD-DCs grown with GM-CSF and salt or BMD-DCs treated with aGM and salt to analyze cellular expression of inflammatory genes. Data are presented as the mean ± SEM and statistical analyses were performed with an unpaired Student’s *t*-test, * *p* < 0.05 vs. Control + GM or Control + aGM; ND = not detectable.

Analysis of aGM-treated BMD-DC populations revealed similar decreases in immune cell subsets, with both BMD-DC phenotypes and their CD38+ subsets showing reductions (Figure 7A,B). Additionally, BMD-cDC2s and CD38+ BMD-cDC2s were decreased further after aGM treatment as well (Figure 7A,B). Further analysis of unsorted BMD-DC populations cultured with GM-CSF and exposed to salt revealed significantly increased expression of *Tnfα*, *Il-6*, and *Il-1β*. However, when BMD-DCs were treated with aGM prior to salt exposure, *Il-6* expression was decreased significantly, while *Tnfα* and *Il-1β* expression returned to baseline (Figure 7C).

When BMDMs were differentiated into DCs with GM-CSF, then exposed to salt and sorted for CD38+ BMD-cDC2s (CD11b+ CD11c+ MHCII+ DCIR2+ CD38+), *Tnfα*, *Il-6*, and *Il-1β* expression were all increased significantly (Figure 7D). Notably, aGM treatment prior to salt exposure significantly reduced *Tnfα* and *Il-1β* expression, while *Il-6* was not detectable, suggesting complete suppression by the inhibitor (Figure 7D). 

## 4. Discussion

GM-CSF is a multifaceted growth factor, and here we highlight its role in SSHTN. Our findings demonstrate that using aGM as a preventive treatment for SSHTN slowed the increase in SBP, reduced renal inflammation, and moderately protected renal function. When aGM was administered as a treatment after the onset of SSHTN, SBP was blunted significantly, with a notable reduction by the end of the model. Renal expression of inflammatory genes was also reduced; however, renal function did not improve significantly, and creatinine clearance was decreased. The lack of renal protection may be attributed to excessive GM-CSF inhibition, reduced blood vasculature, or pre-existing damage caused by L-NAME early in the SSHTN model. GM-CSF plays an important role in tissue repair by supporting cells essential for regeneration and facilitating immune cell recruitment for vascular remodeling. If these processes were excessively inhibited, it could have resulted in an intermediate phenotype rather than a fully protective or detrimental effect. Additionally, because L-NAME is a nitric oxide synthase inhibitor used to induce vascular dysfunction and sensitize mice to salt, it may have caused irreversible baseline damage before GM-CSF modulation was introduced. Further studies are needed to disentangle these effects and determine the optimal balance of GM-CSF modulation in SSHTN.

In our preventive aGM model, we observed decreases in renal Macs (CD45+ CD11b+ CD11c−), CD38+ Macs (CD45+ CD11b+ CD11c− CD38+), M1 Macs, and CD38+ M1 Macs. Additionally, renal pDCs, and CD38+ pDCs were reduced. In the treatment model, renal immune cell populations were differentially affected. Macs (CD45+ CD11b+ F4/80+) CD38+ Macs (CD45+ CD11b+ F4/80+ CD38+), M1 Macs, and CD38+ M1 Macs were decreased significantly. However, DCs were not changed significantly. The M1/M2 ratio is generally considered to be increased in HTN, and flow cytometry analysis revealed that renal M2 macrophages were decreased significantly in the preventative model, but not in the treatment model (Supplementary Figure S3A,B). While we found CD38 to be an adequate marker of activation, traditional activation markers such as CD68 for Macs and CD86 for DCs were also measured to confirm the activated phenotypes (Supplementary Figures S3C,D and S4A,B).

Contrary to expected results, aGM was less effective at inhibiting most DC subset differentiation. We had anticipated that this treatment would significantly affect development, maturation, and survival of DCs; however, it is possible that other cytokines, such as Flt3L, compensated for the absence of GM-CSF. Flt3L-dependent DCs promote T cell activation, and in a Flt3L knockout mouse model of angiotensin II (A2)-induced HTN, classical renal DCs were decreased genetically, and BP was blunted [47]. We had determined previously that cDC2s were of interest; however, they were minimally impacted in vivo, as well as type-1 conventional DCs (cDC1, Supplementary Figure S4C,D). This raises the possibility of a dual treatment targeting both Flt3L and GM-CSF. Nevertheless, aGM was effective at mitigating differentiation of moDCs, pDCs, and CD38+ pDCs in the preventive model. moDCs are also potent activators of T cells, which contribute to the development of full HTN [48]. Depletion of moDCs in the preventive model, along with the resulting blunted SBP response, is a beneficial outcome. Furthermore, pDCs have been reported to play a role in HTN. Srinivas et al. found in a two-kidney one-clip model of renovascular HTN that depleting pDCs with anti-PDCA-1 antibodies resulted in decreased BP, reduced cardiac hypertrophy, decreased lung edema, and diminished oxidative stress [49]. Additionally, we found that renal blood vasculature was increased in historical SSHTN samples. However, when comparing CAB- and aGM-treated mice, renal vasculature was decreased in the aGM group (Supplementary Figure S5A–I).

The effect of aGM on immune cells was assessed directly via the use of BMDMs, BMD-Macs, and BMD-DCs. Previously, we established that BMDMs grown, or “primed”, in GM-CSF exhibited a higher likelihood of trafficking to hypertensive kidneys and differentiating into various innate immune cell phenotypes, including Macs, CD38+ Macs, DCs, and CD38+ DCs [29]. Following adoptive transfer of aGM-treated BMDMs, we observed a significant decrease in the number of cells trafficking to the kidneys as well as a reduction in all Mac and DC phenotypes that are derived from BMDMs. M2 Macs, moDCs, pDCs, and cDC1s, as well as their CD38+/CD68+/CD86+ counterparts, were reduced as well (Supplementary Figure S6A–F).

In vitro treatment with aGM significantly decreased all BMD-Macs, CD38+ BMD-Macs, BMD-DCs, and CD38+ BMD-DC phenotypes we measured. BMD-Macs grown with GM-CSF and treated with salt had increased gene expression of *Tnfα* and *Il-1β*, which was mitigated by treatment with aGM. When sorting for CD38+ BMD-M1 Macs, those grown with GM-CSF and exposed to salt had increased *Tnfα*, *IL-6*, and *IL-1β* expression. Treatment with aGM prior to salt returned *Tnfα* to baseline levels of expression, *Il-6* expression was decreased significantly, and *Il-1β* expression was still increased, but dampened when compared to the GM-CSF-treated cells. BMD-DCs grown with GM-CSF and treated with salt exhibited increased expression of *Tnfα*, *Il-6*, and *lL-1β*. However, treatment with aGM prior to salt mitigated *Tnfα* expression, significantly reduced *Il-6* expression, and returned *Il-1β* expression to baseline. When sorting for CD38+ BMD-cDC2s from GM-CSF-grown BMD-DCs, we observed significantly elevated gene expression of *Tnfα*, *Il-6*, and *Il-1β.* In contrast, when sorting from cells treated with aGM prior to salt, this effectively blocked these increases in *TNFα*, *IL-6*, and *IL-1β* gene expression. The overall ability of aGM to block or blunt pro-inflammatory and pro-hypertensive gene expression further highlights its therapeutic potential in SSHTN. To address potential osmolarity effects, we included mannitol-treated BMD-Macs and BMD-DCs as an osmotic control. Based on these data, Macs were more sensitive to osmotic changes; however, both M1 Macs and activated M1 Macs demonstrated activation through ionic rather than osmotic mechanisms (Supplementary Figure S1A–D). All DC phenotypes appear to be activated primarily via ionic mechanisms (Supplementary Figure S1E–I).

Additionally, we have reported previously immune cell changes in A2-induced HTN. We completed corresponding in vitro studies with A2 and found that aGM in vitro was less effective on BMD-Mac and BMD-DC phenotypes that were treated with A2 (Supplementary Figure S7A–J). Therefore, we did not proceed with in vivo treatments. This suggests either overall mechanistic differences between these two HTN models, dosage differences, or the possibility of incorporating aGM with an already established A2-induced HTN treatment.

A limitation of this study is the use of only female mice for the early model of SSHTN and male mice for the established model of SSHTN when inhibiting GM-CSF. While prior work from our group did not identify significant BP or renal cell immune differences between sexes in this SSHTN model [50], future studies should directly evaluate both to determine sex-specific responses. While we utilized mannitol and salt at the same concentration and saw changes (Supplementary Figure S1A–I), future studies could analyze using double the amount of mannitol. Food and water intake were not monitored in this study, which limits the ability to assess whether changes in BP, renal function, or renal immune cell responses may have been influenced by dietary intake. Future studies incorporating metabolic monitoring systems could help clarify these factors. Flow cytometry gating was used to exclude dead cells from analysis; however, a dedicated live/dead viability assay was not performed, and future studies could incorporate viability assays to confirm that the observed effects are not due to cell stress or death.

This study provides evidence that GM-CSF plays a role in SSHTN. Our data demonstrate that inhibiting GM-CSF, whether as a preventative or therapeutic approach, lowers BP, reduces renal inflammatory gene expression, alters renal function, and differentially affects renal immune cell phenotypes. We also found that BMDMs treated with aGM were less likely to traffic to SSHTN kidneys or differentiate into hallmark immune cells of HTN. Additionally, both of our previously identified immune cells of interest, CD38+ M1 Macs and CD38+ cDC2s, exhibited increased pro-inflammatory gene expression, which was dampened by aGM treatment. Further studies are underway to better define the role of GM-CSF in HTN.

## Data Availability

All supporting data are included within the article.

## Supplementary Materials

The following supporting information can be downloaded at https://www.mdpi.com/article/10.3390/cells14151144/s1, Supplementary Table S1: Antibody panels used on murine kidneys and cells; Supplementary Table S2: Primer sequences for qRT-PCR analysis of murine renal tissue and immune cells; Supplementary Figure S1: Mannitol osmolarity control for BMD-Macs and BMD-DCs; Supplementary Figure S2: Flow cytometry gating strategies for innate immune cells; Supplementary Figure S3: M2 Mac and CD68+ Mac phenotypes from the preventive and treatment aGM models were affected differentially; Supplementary Figure S4: aGM treatment was less effective on DC subsets from both the preventive and treatment SSHTN models; Supplementary Figure S5: Renal blood vessel counts; Supplementary Figure S6: Adoptive transfer of CellTracker+ BMDM alternative phenotypes; Supplementary Figure S7: aGM was less effective at reducing the effects of A2 on BMD-Macs and BMD-DCs.

## Abbreviations

The following abbreviations are used in this manuscript:

A2	Angiotensin II
aGM	Anti-granulocyte-macrophage colony-stimulating factor
BMD	Bone marrow derived
BMD-cDC2	Bone marrow derived type-2 conventional dendritic cells
BMD-DC	Bone marrow derived dendritic cells
BMDMs	Bone marrow derived monocytes
BMD-Macs	Bone marrow derived macrophages
BMD-M1 Macs	Bone marrow derived M1 macrophages
BP	Blood pressure
CAB	Control antibody
cDC1	Type-1 conventional dendritic cells
cDC2s	Type-2 conventional dendritic cells
CD38	Cluster of differentiation 38
DCs	Dendritic cells
FENa	Fractional excretion of sodium
GM-CSF	Granulocyte-macrophage colony-stimulating factor
IL	Interleukin
Mac	Macrophage
moDCs	Monocyte derived dendritic cells
NAD+	Nicotinamide adenine dinucleotide
pDCs	Plasmacytoid DCs
SBP	Systolic blood pressure
SSHTN	Salt-sensitive hypertension
TNFα	Tumor necrosis factor alpha
