# Supplementary material for: Granulocyte-Macrophage Colony-Stimulating Factor Inhibition Ameliorates Innate Immune Cell Activation, Inflammation, and Salt-Sensitive Hypertension
Source: Cells. 2025 Jul 24;14(15):1144. doi: 10.3390/cells14151144 (PMC12345731; doi:10.3390/cells14151144)
Supplement: Supplementary file 1 [file cells-14-01144-s001.zip › cells-3752862-supplementary.pdf]

(A)

Panel	Macrophages Flow Antibody Panel							
Fluorochrome	APC	APC-Cy7	BV421	BV711	BV785	FITC	PE-Cy7	Ghost Dye 710
Antigen	CD11c	CD38	CD45.2	CD68	CD206	CD11b	F4/80	LIVE/DEAD
Dilution Factor	1:100 for Kidneys; 1:200 for Cultured Cells							1:400
Clone	N418	90	104	FA-11	C068C2	M1/70	BM8	
Manufacturer	BioLegend	BioLegend	BD	BioLegend	BioLegend	BioLegend	BIoLegend	Tonbo

(B)

Panel	Dendritic Cells Flow Antibody Panel										
Fluorochrome	APC	APC-Cy7	BUV395	BUV496	BUV737	BV421	BV605	BV650	FITC	PerCP-eFluor ™ 710	Ghost Dye 710
Antigen	CD11c	CD38	Siglec-H	CD86	Ly-6C	CD45.2	XCR1	MHCII	CD11b	DCIR2	LIVE/DEAD
Dilution Factor	1:100 for Kidneys; 1:200 for Cultured Cells										1:400
Clone	N418	90	551	PO3	HK1.4rMAb	104	ZET	M5/114.15.2	M1/70	33D1	
Manufacturer	BioLegend	BioLegend	BD Biosciences	BD Biosciences	BD Biosciences	BD	BioLegend	BioLegend	BioLegend	Thermo Fisher Scientific	Tonbo

(C)

Panel	Adoptive Transfer Flow Cytometry Antibody Panel							
Fluorochrome	APC	APC-Cy7	BV421	BV785	FITC	PE-Cy7	CellTracker™ Deep Red Dye	Ghost Dye 710
Antigen	CD11c	CD38	CD45.2	CD206	CD11b	F4/80		LIVE/DEAD
Dilution Factor	1:100 for Kidneys							1:400
Clone	N418	90	104	C068C2	M1/70	BM8		
Manufacturer	BioLegend	BioLegend	BD	BioLegend	BioLegend	BIoLegend	Thermo Fisher Scientific	Tonbo

(D)

Panel	CD38+ M1 Macrophage Cell Sort				
Fluorochrome	APC	APC-Cy7	PE	FITC	Ghost Dye 510
Antigen	CD11c	CD38	CD206	CD11b	LIVE/DEAD
Dilution Factor	1:200 for Cultured Cells				1:400
Clone	N418	90	C068C2	M1/70	
Manufacturer	BioLegend	BioLegend	BioLegend	BioLegend	Tonbo

(E)

Panel	CD38+ cDC2 Cell Sort					
Fluorochrome	APC	APC-Cy7	BV650	FITC	PerCP-eFluor ™ 710	Ghost Dye 510
Antigen	CD11c	CD38	MHCII	CD11b	DCIR2	LIVE/DEAD
Dilution Factor	1:200 for Cultured Cells					1:400
Clone	N418	90	M5/114.15.2	M1/70	33D1	
Manufacturer	BioLegend	BioLegend	BioLegend	BioLegend	Thermo Fisher Scientific	Tonbo

Supplementary Table S1. Antibody panels used on murine kidneys and cells. (A) Mac flow cytometry antibody panel: this panel was used to identify Mac subtypes in vivo and in vitro; (B) DC flow cytometry antibody panel: this panel was used to identify DC subtypes in vivo and in vitro; (C) adoptive transfer innate immune cell flow cytometry antibody panel: this panel was used to identify the BMDMs that were adoptive transferred into the hypertensive mice as well as determine what the adoptive transferred cells turned into; (D) CD38+ M1 Mac cell sort: this antibody panel was used to distinguish and sort specifically for CD38+ M1 Macs out of the BMD-Macs in vitro; (E) CD38+ cDC2 cell sort: this antibody panel was used to distinguish and sort specifically for CD38+ cDC2s out of the BMD-DCs in vitro. Abbreviations: APC = allophycocyanin; FITC = fluorescein isothiocyanate; BUV395= Brilliant Ultraviolet; BV421= Brilliant Violet; PE = phycoerythrin; PerCP-Cy5.5 = peridinin chlorophyll protein complex cyanine 5.5

Target	Forward (5' to 3')	Reverse (5' to 3')
Tnfa	GAGAAAGTCAACCTCCTCTCTG	GAAGACTCCTCCCAGGTATATG
Il-6	GAGGATACCACTCCCAACAGACC	AAGTGCATCATCGTTGTTCATA
Il-1b	GCCACCTTTTGACAGTGATGAG	GACAGCCCAGGTCAAAGGTT
RPS18	CATGCAGAACCCACGACAGTA	CCTCACGCAGCTTGTTGTCTA

Supplementary Table S2. Primer sequences for qRT-PCR analysis of murine renal tissue and immune cells. All sequences were verified through National Center for Biotechnology Information Primer-BLAST and single products were confirmed with a melting point dissociation step post amplification. Abbreviations: Tnfa, tumor necrosis factor alpha; Il-6, interleukin 6; Il-1b, interleukin 1 beta; RPS18, ribosomal protein subunit 18.

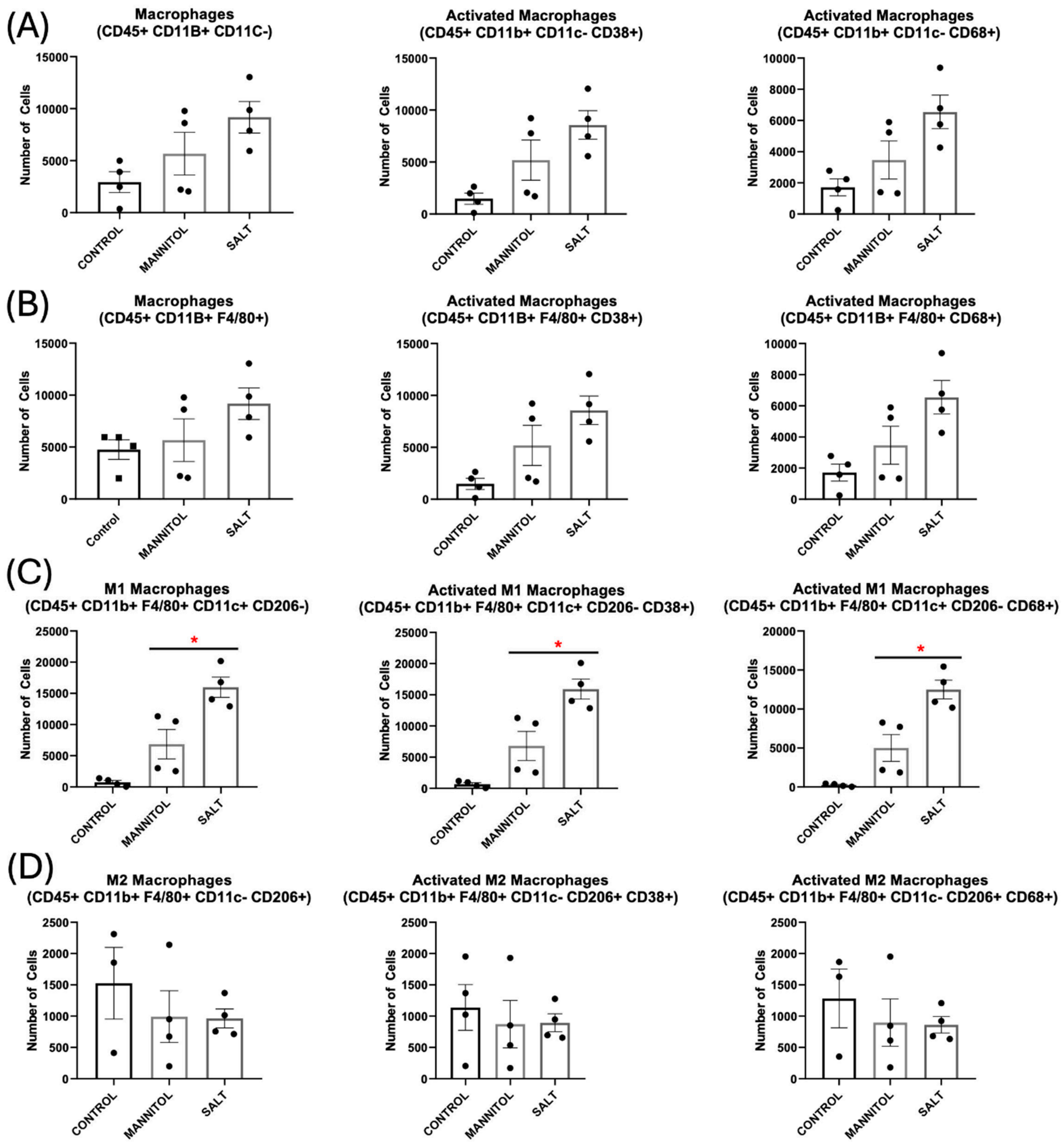

(A)
Macrophages
(CD45+ CD11B+ CD11C-)
Activated Macrophages
(CD45+ CD11b+ CD11c- CD38+)
Activated Macrophages
(CD45+ CD11b+ CD11c- CD68+)
(B)
Macrophages
(CD45+ CD11B+ F4/80+)
Activated Macrophages
(CD45+ CD11B+ F4/80+ CD38+)
Activated Macrophages
(CD45+ CD11B+ F4/80+ CD68+)
(C)
M1 Macrophages
(CD45+ CD11b+ F4/80+ CD11c+ CD206-)
Activated M1 Macrophages
(CD45+ CD11b+ F4/80+ CD11c+ CD206- CD38+)
Activated M1 Macrophages
(CD45+ CD11b+ F4/80+ CD11c+ CD206- CD68+)
(D)
M2 Macrophages
(CD45+ CD11b+ F4/80+ CD11c- CD206+)
Activated M2 Macrophages
(CD45+ CD11b+ F4/80+ CD11c- CD206+ CD38+)
Activated M2 Macrophages
(CD45+ CD11b+ F4/80+ CD11c- CD206+ CD68+)
Number of Cells
CONTROL
Control
MANNITOL
SALT
*

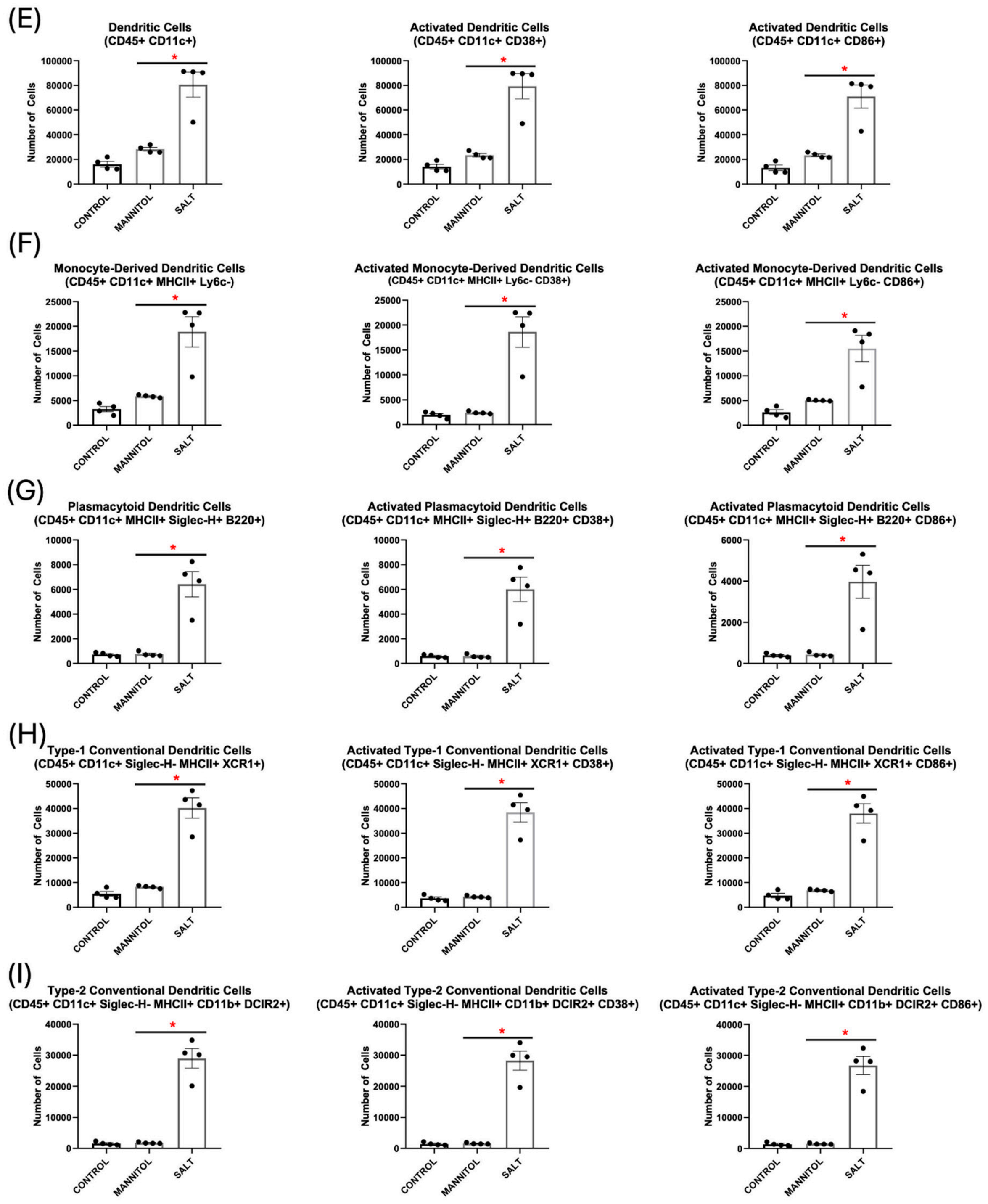

Supplementary Figure S1. Mannitol osmolarity control for BMD-Macs and BMD-DCs. Via flow cytometry control (normal salt), mannitol, and salt (high salt, 180 mM) treatments were compared assessing osmotic or ionic activation on **(A)** Macs and activated Macs, **(B)** Macs and activated Macs, **(C)** M1 Macs and activated M1 Macs, **(D)** M2 Macs and activated M2 Macs, **(E)** DCs and activated DCs, **(F)** moDCs and activated moDCs, **(G)** pDCs and activated pDCs, **(H)** cDC1s and activated cDC1s, and **(I)** cDC2s and activated cDC2s. Data are presented as the mean ± SEM and statistical analyses were performed with an unpaired Student's t-test, *$p < 0.05$ mannitol vs salt.

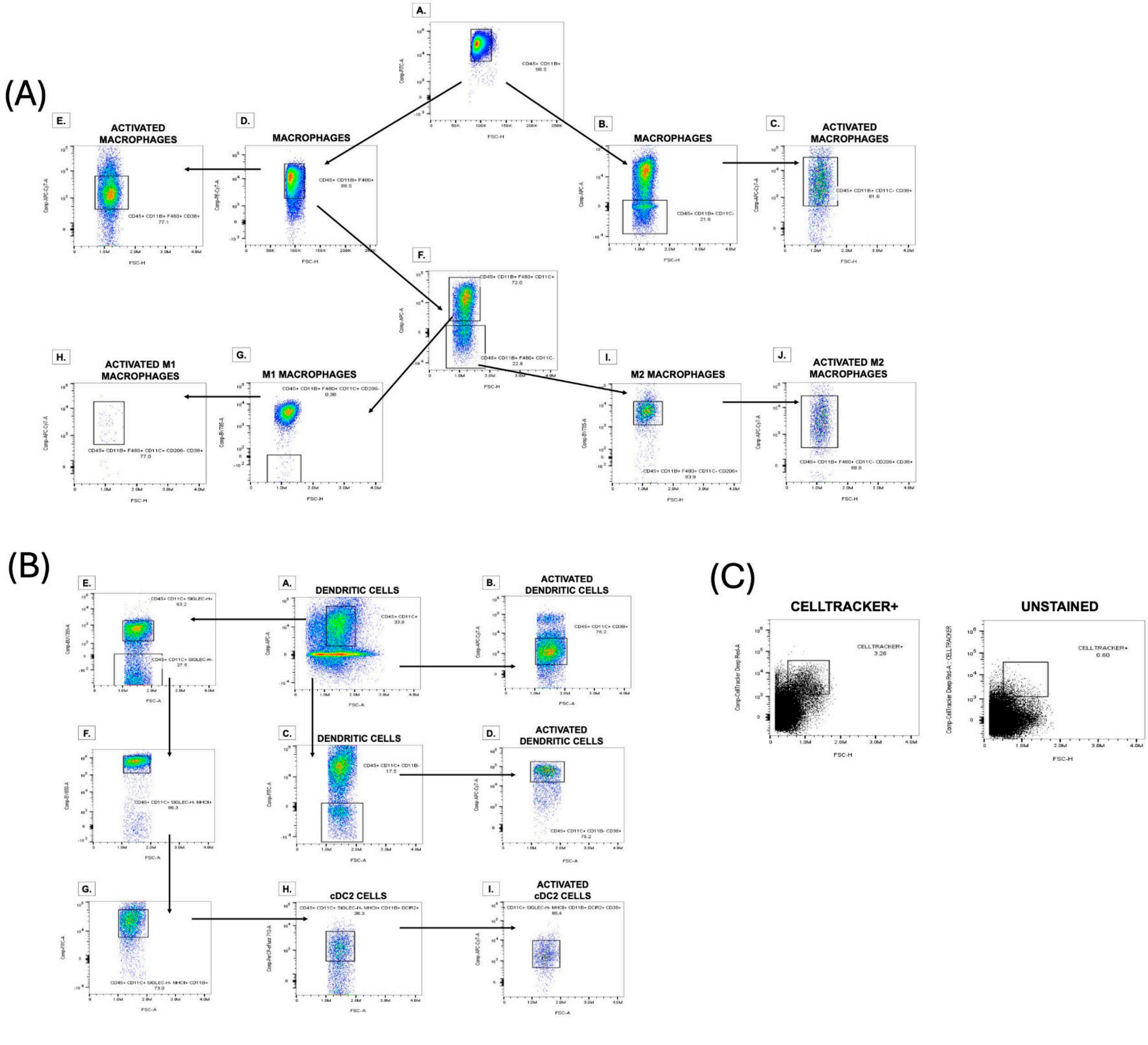

(A)
A.
CD45+ CD11B+
98.5
B.
MACROPHAGES
CD45+ CD11B+ CD11C-
21.6
C.
ACTIVATED MACROPHAGES
CD45+ CD11B+ CD11C- CD38+
81.6
D.
MACROPHAGES
CD45+ CD11B+ F480+
88.5
E.
ACTIVATED MACROPHAGES
CD45+ CD11B+ F480+ CD38+
77.1
F.
CD45+ CD11B+ F480+ CD11C+
72.0
CD45+ CD11B+ F480+ CD11C-
22.8
G.
M1 MACROPHAGES
CD45+ CD11B+ F480+ CD11C+ CD206-
0.38
H.
ACTIVATED M1 MACROPHAGES
CD45+ CD11B+ F480+ CD11C+ CD206- CD38+
77.0
I.
M2 MACROPHAGES
CD45+ CD11B+ F480+ CD11C- CD206+
83.9
J.
ACTIVATED M2 MACROPHAGES
CD45+ CD11B+ F480+ CD11C- CD206+ CD38+
88.9
(B)
A.
DENDRITIC CELLS
CD45+ CD11C+
33.8
B.
ACTIVATED DENDRITIC CELLS
CD45+ CD11C+ CD38+
76.2
C.
DENDRITIC CELLS
CD45+ CD11C+ CD11B-
17.5
D.
ACTIVATED DENDRITIC CELLS
CD45+ CD11C+ CD11B- CD38+
78.2
E.
CD45+ CD11C+ SIGLEC-H+
63.2
CD45+ CD11C+ SIGLEC-H-
27.5
F.
CD45+ CD11C+ SIGLEC-H- MHCII+
86.3
G.
CD45+ CD11C+ SIGLEC-H- MHCII+ CD11B+
73.0
H.
cDC2 CELLS
CD45+ CD11C+ SIGLEC-H- MHCII+ CD11B+ DCIR2+
38.3
I.
ACTIVATED cDC2 CELLS
CD11C+ SIGLEC-H- MHCII+ CD11B+ DCIR2+ CD38+
85.4
(C)
CELLTRACKER+
CELLTRACKER+
3.26
UNSTAINED
CELLTRACKER+
0.60
FSC-H
FSC-A

(D)

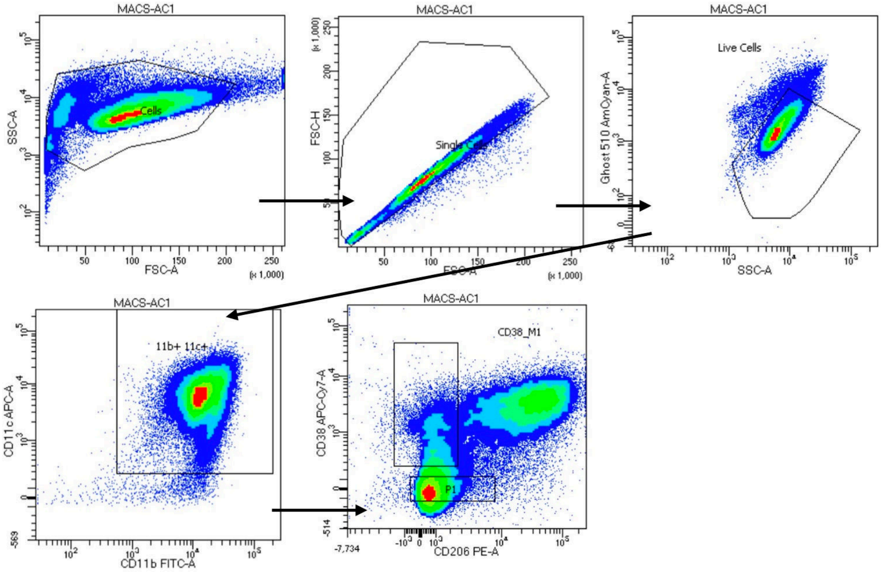

(E)

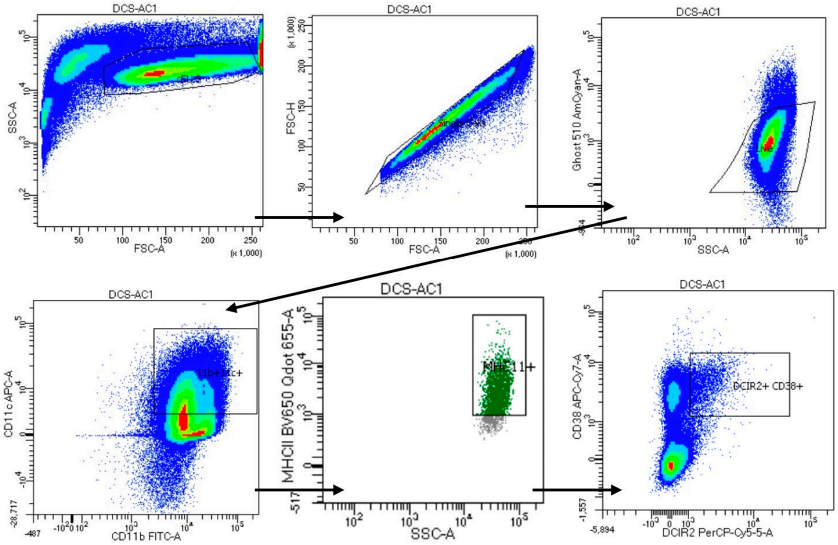

Supplementary Figure S2. Flow cytometry gating strategies for innate immune cells. All flow cytometry panels were initially gated with a live dead gate, single cell gate, and CD45+ gate before proceeding to the first gate of each strategy. **(A)** Gating strategy for Macs/activated Macs: **A.** CD45+ CD11b+ **B.** CD45+ CD11b+ CD11c- **C.** CD45+ CD11b+ CD11c- CD38+ OR **A.** CD45+ CD11b+ **D.** CD45+ CD11b+ F4/80+ **E.** CD45+ CD11b+ F4/80+ CD38+; gating strategy for M1 Macs/activated M1 Macs: **A.** CD45+ CD11b+ **D.** CD45+ CD11b+ F4/80+ **F.** CD45+ CD11b+ F4/80+ CD11c+ CD11c- **G.** CD45+ CD11b+ F4/80+ CD11c+ CD206- **H.** CD45+ CD11b+ F4/80+ CD11c+ CD206- CD38+; gating strategy for M2 Macs/activated M2

Macs: **A.** CD45+ CD11b+ **D.** CD45+ CD11b+ F4/80+ **F.** CD45+ CD11b+ F4/80+ CD11c+ CD11c- **I.** CD45+ CD11b+ F4/80+ CD11c- CD206+ **J.** CD45+ CD11b+ F4/80+ CD11c- CD206+ CD38+. **(B)** Gating strategy for DCs/activated DCs: **A.** CD45+ CD11c+ **B.** CD45+ CD11c+ CD38+ OR **A.** CD45+ CD11c+ **C.** CD45+ CD11c+ CD11b- **D.** CD45+ CD11c+ CD11b- CD38+; gating strategy for cDC2s/activated cDC2s: **A.** CD45+ CD11c+ **E.** CD45+ CD11c+ Siglec-H- **F.** CD45+ CD11c+ Siglec-H- MHCII+ **G.** CD45+ CD11c+ Siglec-H- MHCII+ CD11b+ **H.** CD45+ CD11c+ Siglec-H- MHCII+ CD11b+ DCIR2+ **I.** CD45+ CD11c+ Siglec-H- MHCII+ CD11b+ DCIR2+ CD38+. **(C)** Gating strategy for adoptive transfer CellTracker+ cells: Mac/activated Mac/DC/activated DC gating strategies were used, however, CellTracker+ cells were gated prior to these gates. **(D)** Gating strategy for cell sorting isolation of CD38+ M1 Macs: **A.** Cells **B.** Single cells **C.** Live cells **D.** CD11b+ CD11c+ **E.** CD11b+ CD11c+ CD206- CD38+**.** **(E)** Gating strategy for cell sorting isolation of CD38+ cDC2s: **A.** Cells **B.** Single cells **C.** Live cells **D.** CD11b+ CD11c+ **E.** CD11b+ CD11c+ MHCII+ **F.** CD11b+ CD11c+ MHCII+ DCIR2+ CD38+.

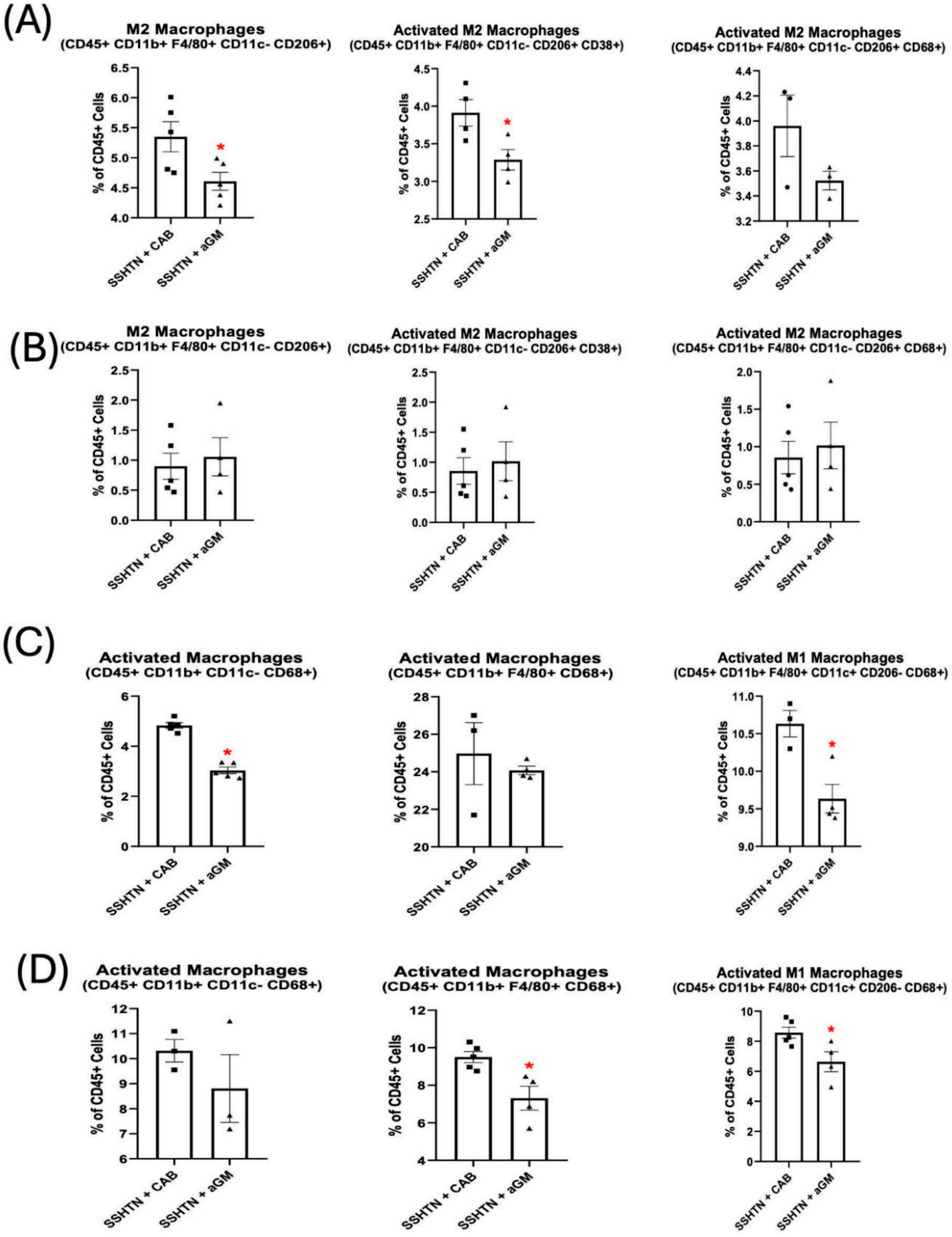

Supplementary Figure S3. M2 Mac and CD68+ Mac phenotypes from the preventive and treatment aGM models were affected differentially. Via flow cytometry, renal M2 Macs and CD38+ M2 Macs were analyzed in the **(A)** preventive aGM model as well as the **(B)** treatment model. A traditional CD68+ gate was used as a confirmatory gate for Mac activation in the **(C)** preventive model as well as the **(D)** treatment model.

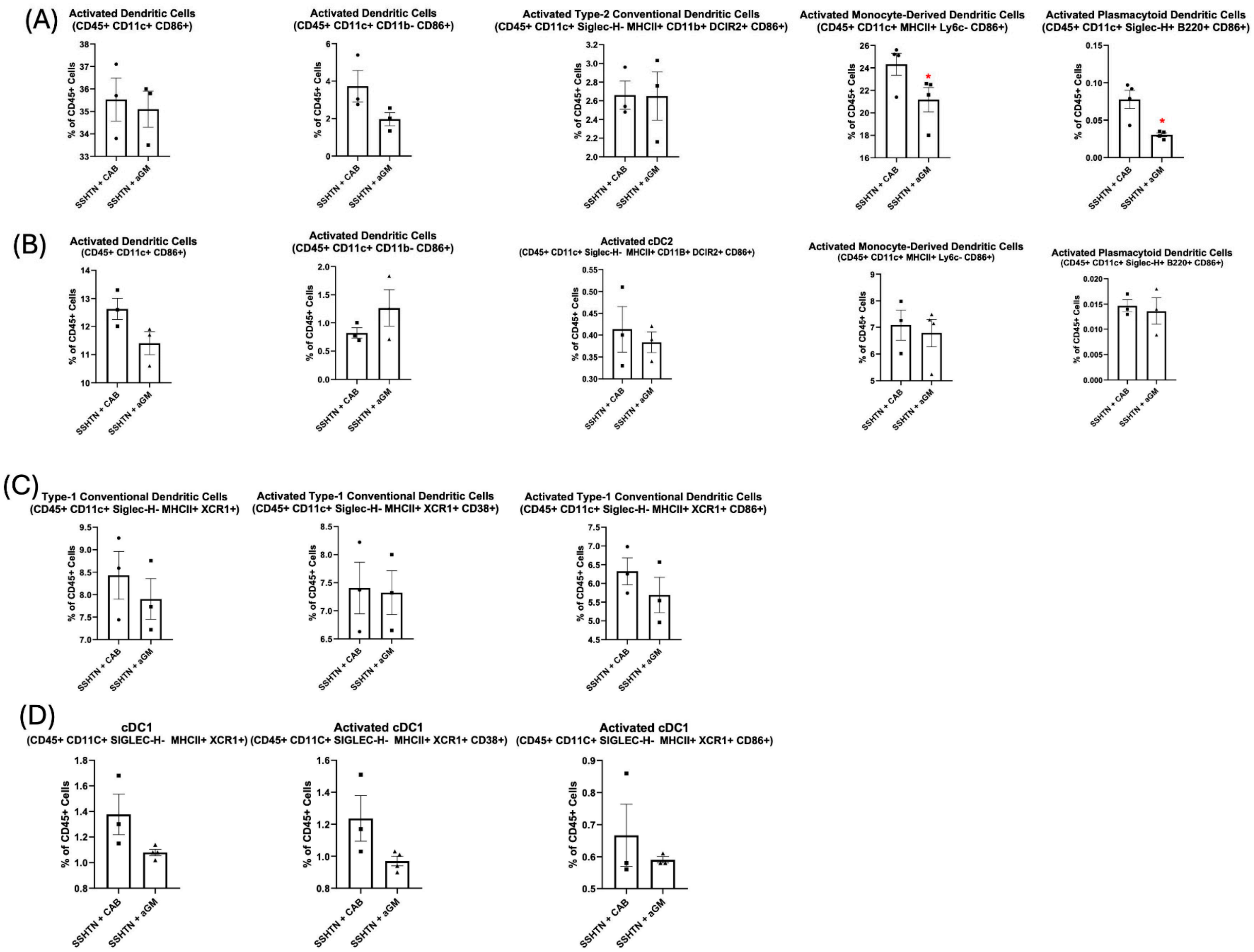

Supplementary Figure S4. aGM treatment was less effective on DC subsets from both the preventive and treatment SSHTN models. Via flow cytometry, analysis with a traditional CD86+ gate was used to confirm DC activation in the **(A)** preventive model as well as the **(B)** treatment model. Another immune cell of interest, cDC1 was analyzed in both the aGM preventive **(C)** and aGM treatment **(D)** SSHTN models.

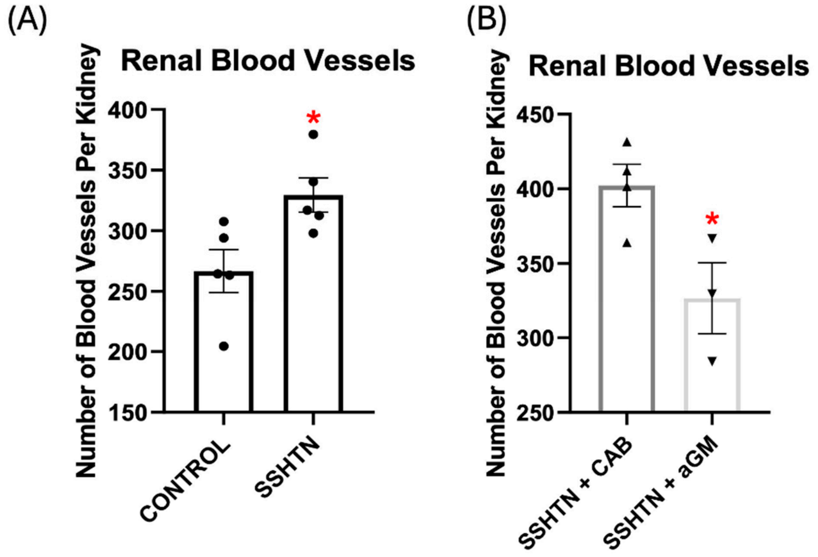

Supplementary Figure S5. Renal blood vessel counts. **(A)** The number of blood vessels was counted in historical control vs SSHTN renal tissue samples. **(B)** The number of blood vessels was counted in SSHTN+ CAB vs SSHTN + aGM renal tissue. Data are presented as the mean ± SEM and statistical analyses were performed with an unpaired Student's t-test, *$p < 0.05$ vs. control or *$p < 0.05$ vs. SSHTN + CAB.

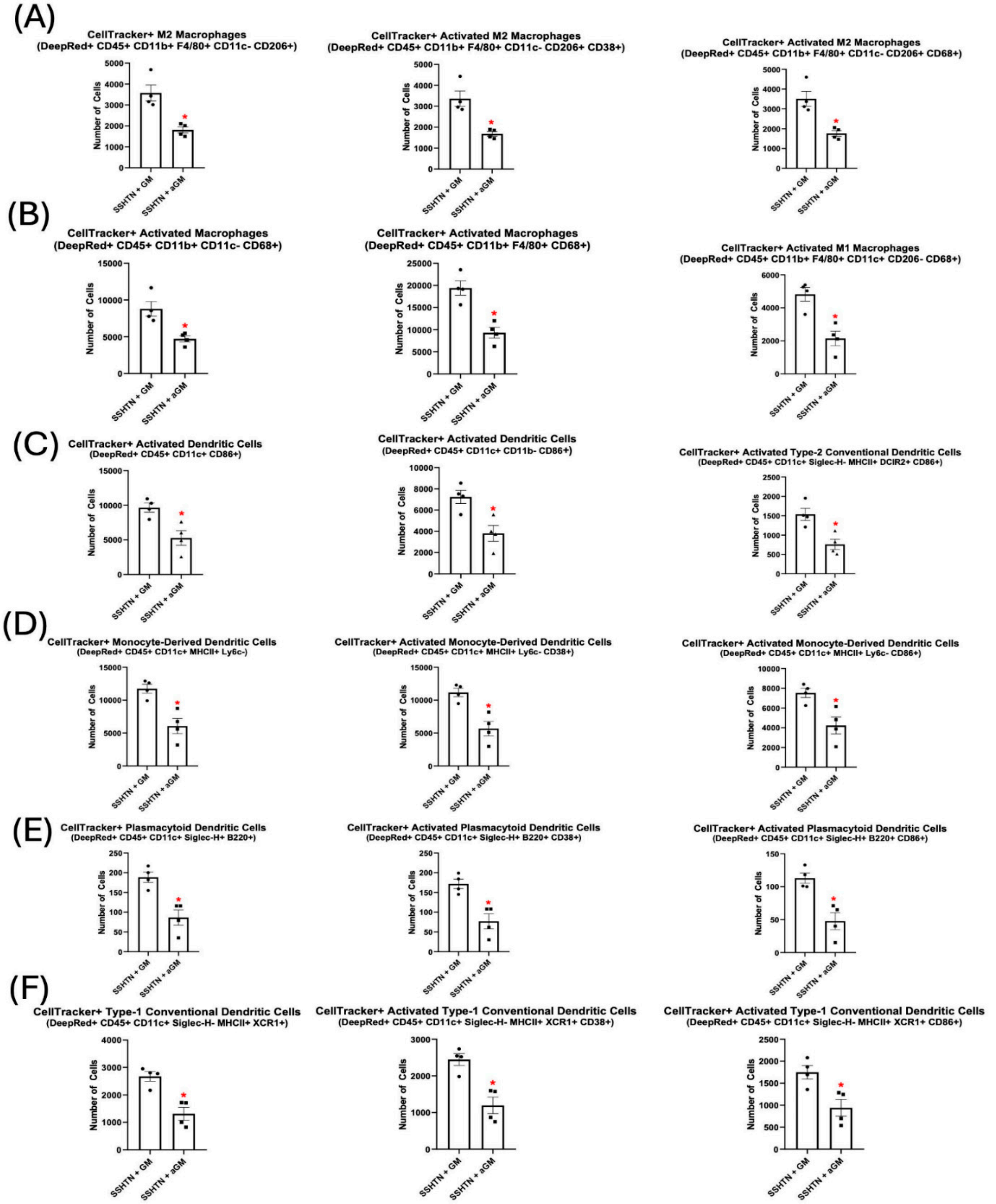

Supplementary Figure S6. Adoptive transfer of CellTracker+ BMDM alternative phenotypes. Populations of CellTracker+ **(A)** M2 Macs, 38+ M2 Macs, CD68+ M2 Macs **(B)** CD11c- CD68+ Macs, F4/80+ CD68+ Macs, CD68+ M1 Macs **(C)** CD11c+ CD86+ DCs, CD11b- CD86+ DCs, CD86+ cDC2s **(D)** moDC, CD38+ moDC, CD86+ moDC **(E)** pDC, CD38+ pDC, CD86+ pDC **(F)** cDC1, CD38+ cDC1, CD86+ cDC1.

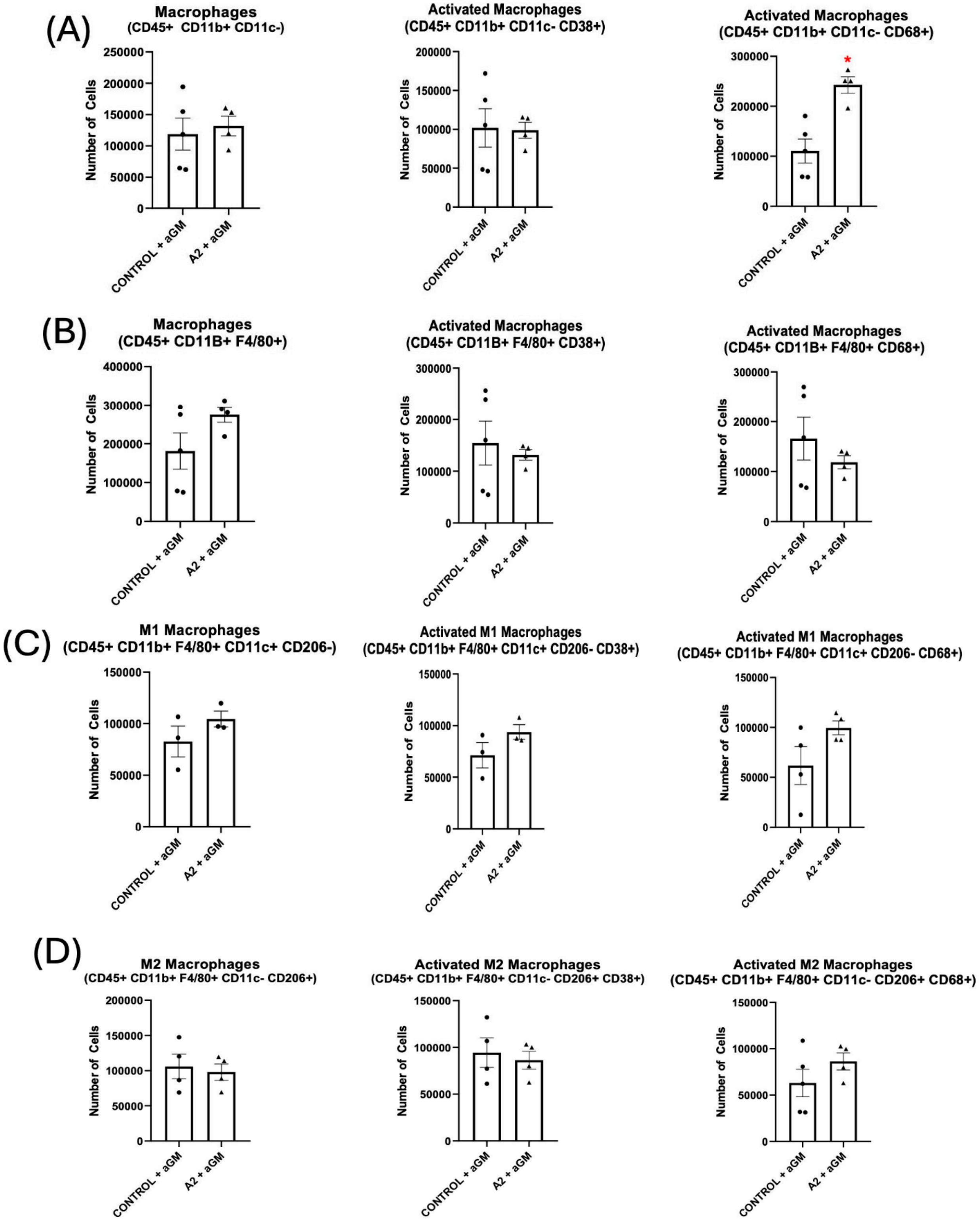

(A)
Macrophages
(CD45+ CD11b+ CD11c-)
Activated Macrophages
(CD45+ CD11b+ CD11c- CD38+)
Activated Macrophages
(CD45+ CD11b+ CD11c- CD68+)
(B)
Macrophages
(CD45+ CD11B+ F4/80+)
Activated Macrophages
(CD45+ CD11B+ F4/80+ CD38+)
Activated Macrophages
(CD45+ CD11B+ F4/80+ CD68+)
(C)
M1 Macrophages
(CD45+ CD11b+ F4/80+ CD11c+ CD206-)
Activated M1 Macrophages
(CD45+ CD11b+ F4/80+ CD11c+ CD206- CD38+)
Activated M1 Macrophages
(CD45+ CD11b+ F4/80+ CD11c+ CD206- CD68+)
(D)
M2 Macrophages
(CD45+ CD11b+ F4/80+ CD11c- CD206+)
Activated M2 Macrophages
(CD45+ CD11b+ F4/80+ CD11c- CD206+ CD38+)
Activated M2 Macrophages
(CD45+ CD11b+ F4/80+ CD11c- CD206+ CD68+)
Number of Cells
CONTROL + aGM
A2 + aGM

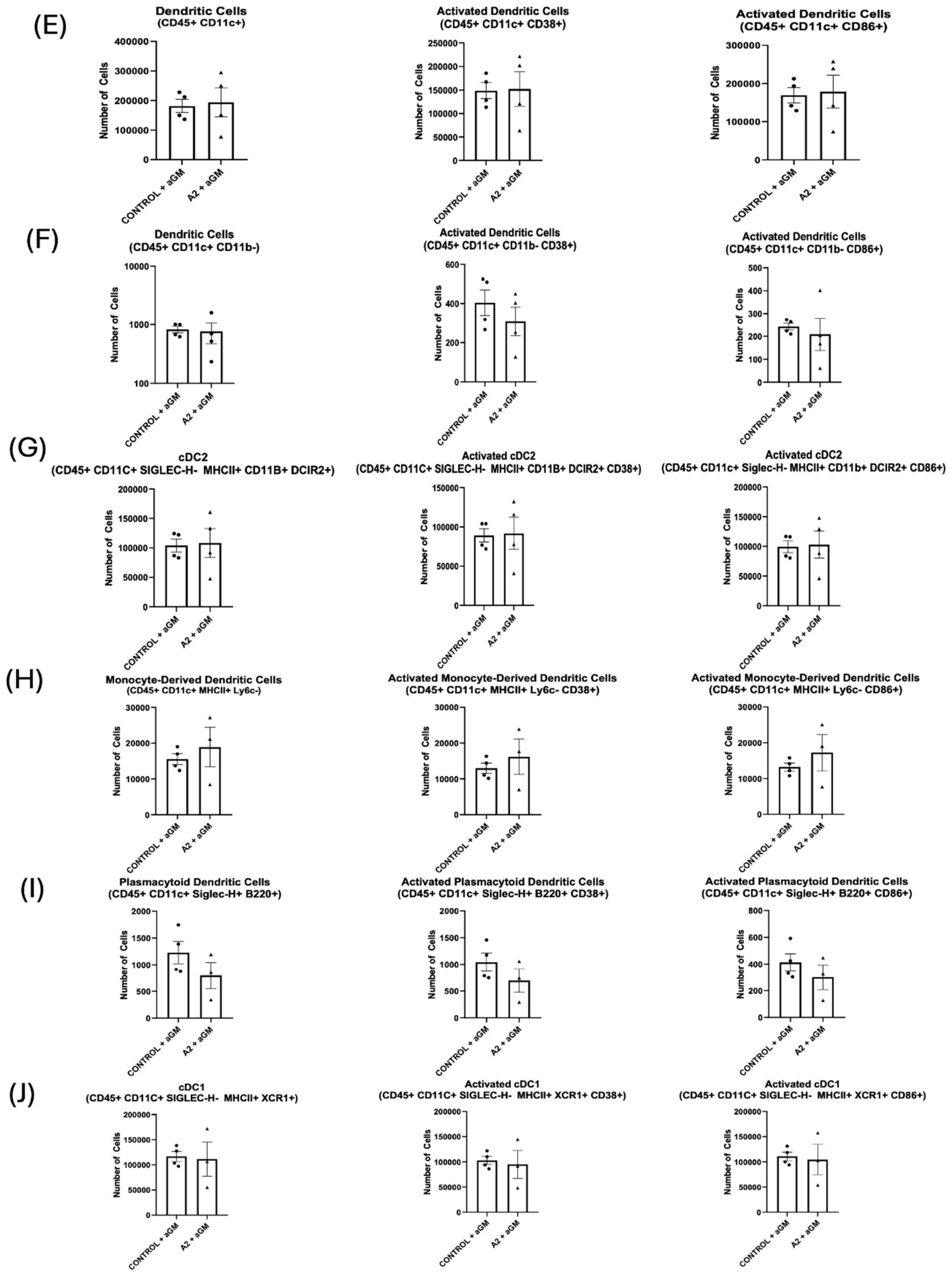

Supplementary Figure S7. aGM was less effective at reducing the effects of A2 on BMD-Macs and BMD-DCs. Populations of **(A)** 11c- Macs, 11c- CD38+ Macs, 11c- CD68+ Macs **(B)** F4/80+ Macs, F4/80+ CD38+ Macs, F4/80+ CD68+ Macs **(C)** M1 Macs, CD38+ M1 Macs, CD68+ M1 Macs **(D)** M2 Macs, CD38+ M2 Macs, CD68+ M2 Macs **(E)** CD11c+ DCs, CD11c+ CD38+ DCs, CD11c+ CD86+ DCs **(F)** CD11b- DCs, CD11b- CD38+ DCs, CD11b- CD86+ DCs **(G)** cDC2, CD38+ cDC2, CD86+ cDC2s (**H)** moDC, CD38+ moDC, CD86+ moDC **(I)** pDC, CD38+ pDC, CD86+ pDC **(J)** cDC1, CD38+ cDC1, CD86+ cDC1.
